# Individual identifiability following Procrustes alignment of functional gradients: effect of subspace dimensionality

**DOI:** 10.1038/s42003-025-09509-3

**Published:** 2026-01-10

**Authors:** Leonard Sasse, Casey Paquola, Juergen Dukart, Felix Hoffstaedter, Simon B. Eickhoff, Kaustubh R. Patil

**Affiliations:** 1 Institute of Neuroscience and Medicine, Brain and Behaviour (INM-7), Research Centre Jülich, Jülich, Germany; 2 Institute of Systems Neuroscience, Medical Faculty, Heinrich-Heine-University Düsseldorf, Düsseldorf, Germany; 3 Max Planck School of Cognition, Leipzig, Germany

**Keywords:** Network models, Data processing

## Abstract

Functional connectivity (FC) gradients derived from fMRI provide valuable insights into individual differences in brain organisation, yet aligning these gradients across individuals poses challenges for meaningful group comparisons. Procrustes alignment is often employed to standardize gradients, but the choice of the number of gradients used in alignment introduces complexities that may affect the validity of individual-level analyses. In this study, we systematically investigate the impact of varying gradient counts in Procrustes alignment on the principal FC gradient, using data from four high-quality fMRI datasets, including the Human Connectome Project (HCP-YA), Amsterdam Open MRI Collection (AOMIC) PIOP1 and PIOP2, and Cambridge Centre for Ageing and Neuroscience (Cam-CAN). We find that increasing the number of gradients used in alignment enhances subject identification. To further probe these effects, we use machine learning to predict fluid intelligence and age, and a motion prediction analysis, revealing that higher alignment gradient counts may introduce information from lower gradients into the principal gradient with implications for the interpretation of individual-level analyses.

## Abstract (web summary)

Systematic evaluation of Procrustes alignment shows how using more functional gradients alters principal gradients and affects identifiability, highlighting key implications for individual-level fMRI analyses.

## Introduction

Functional connectivity (FC) derived using functional magnetic resonance imaging (fMRI) is a cornerstone of research for unravelling human brain organisation^1–3^. By examining the temporal relationships between different brain regions, FC analysis has helped unveil intricate networks that underlie various cognitive processes and behaviours^4,5^. FC is also commonly used for investigating individual differences in brain-behaviour associations^6–11^. Beyond traditional FC analysis, more abstract representations such as functional gradients have gained popularity^12–14^ for their ability to reveal systematic patterns of connectivity across the brain^15,16^. The “principal gradient” represents the primary axis of variance from unimodal sensorimotor cortical areas towards heteromodal association cortices, thereby resembling the hierarchical organisation of the cortex identified in prior anatomical work^15,17^. Notably, gradients have shown some potential in the study of inter-individual differences as a reliable and predictive feature^18–22^. For instance, individual differences in functional gradients have been related to variables of clinical interest, such as age or autism diagnosis^23–25^.

When gradients are computed from FC matrices, each parcel (distinct region of the brain) is assigned a value along the gradient. These values can vary in sign across individuals, meaning that the direction of the gradient can be flipped. Additionally, gradients are ordered based on their eigenvalues, but this ordering of gradients can also differ between individuals. Therefore, the gradients need to be aligned to make them comparable. Procrustes alignment is often employed for this purpose^16,20,26^. Procrustes alignment aims to bring the gradients from different individuals into a common space using a group-level gradient as a reference, thereby simplifying group-level analyses^23,27^.

When performing Procrustes alignment one can choose the number of gradients to align simultaneously. Although there is no formal default or theoretical consensus, it has become common practice in the literature to use 10 gradients for alignment, which has shown to maximise the fit between the group- and individual-level gradients^28,29^. Ten is also the default number for gradient extraction in the most commonly used gradients toolbox, BrainSpace (version 0.1.10^16^). The gradient extraction and alignment can conveniently be performed in one step using the “.fit()” method of the GradientMaps object. However, it is common to restrict the downstream analysis only to the first few gradients, for instance, three, arguably because some biological interpretation can be attributed to them^15,19,25,30–34^. Given Procrustes alignment transforms each gradient based on information from all the gradients used in the alignment procedure, the number of gradients used for alignment can considerably impact the outcome. Therefore, understanding the impact of this choice in empirical research is crucial. Further, if Procrustes alignment does use information obtained from all the gradients to transform a specific gradient (e.g., the principal gradient is transformed using all 10 gradients used in alignment), it is critical to understand the origin and nature of the signal used in this process. This is especially critical if the transformation captures noise or relies on unwanted signals. For example, motion signals can persist in FC data even after extensive processing independent of the specific denoising pipeline chosen^35–39^.

Individual-level analysis is a promising and highly anticipated application of FC and, by extension, of FC gradients^22^. However, the impact of Procrustes alignment on individual-level analysis, such as machine-learning-based prediction has not been fully accessed. As different individuals need alignment to a different extent, this consideration becomes crucial when investigating associations of specific gradients with individual-level behaviour, cognition and disease status. It is possible that individual characteristics might affect the alignment, and if so, then which individual characteristics contribute to the extent of the required transformations has not been explored. Specifically, using a higher number of gradients in the alignment process allows for a greater capacity to fit the data. As a result, there are two possibilities: the Procrustes transformation may leverage this additional capacity to minimise individual-level variance, potentially erasing meaningful subject-specific information, or it may inadvertently introduce variance from additional gradients back into the principal gradient. In either case, the number of gradients used in the alignment meaningfully affects the nature and outcome of the transformation. Clarifying those questions is therefore essential for establishing gradients as a biomarker for individual-level analysis.

Here, we investigate the impact of the number of gradients used in Procrustes alignment on the aligned principal gradient, and its use in subject-level downstream analyses across different datasets including the high quality resting fMRI data available in the Human Connectome Project (HCP-YA) S1200 dataset^40^, as well as resting state fMRI data from the Amsterdam Open MRI Collection (AOMIC) and the Cambridge Centre for Ageing and Neuroscience (Cam-CAN). In particular, we test for the impact of motion on the magnitude of the Procrustes transformation and its relationship to the number of gradients used in alignment. In addition, we also investigate the impact of Procrustes alignment on the principal gradients capacity to predict fluid intelligence and age using a machine learning approach.

## Results

### Identification and differential identifiability

First, we explored the impact of the number of gradients used in Procrustes alignment on subject-level analyses by evaluating two key metrics: identification accuracy and differential identifiability (see “Methods”–“Identification and Differential Identifiability”). These metrics provide insight into how well the aligned principal gradient can capture individual-level differences. Identification accuracy refers to the proportion of participants who were matched with highest correlation between two fMRI sessions based on their principal gradient. Differential identifiability measures the difference between the mean within-subject correlations and the mean between-subject correlations. Importantly, in all our downstream analyses, we only use the first (“principal”) gradient, independent of how many gradients were used in Procrustes alignment.

**Fig. 1:**
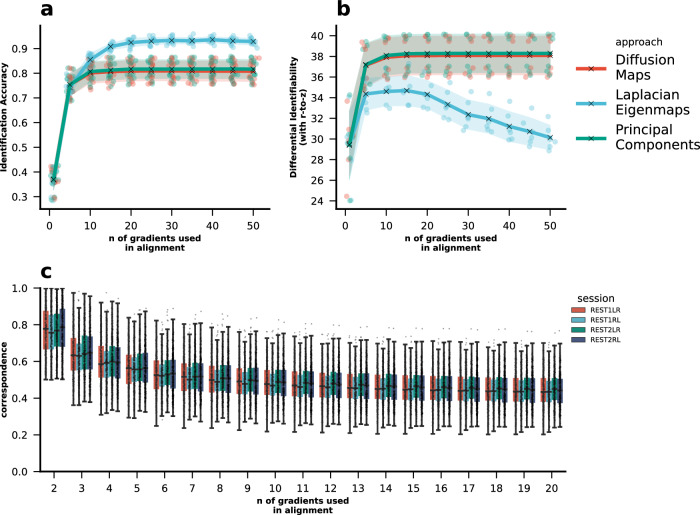
Impact of Procrustes alignment on subject specificity of FC gradients. Impact of Procrustes alignment on **a** identification accuracy and **b** differential identifiability. For each subject, gradients were extracted per session (kernel = normalized_angle; sparsity = 0.9). They were then aligned to the holdout reference gradient using Procrustes alignment. Identification accuracy and differential identifiability were calculated for each combination of sessions (${N}_{{Sessions}}=\,4$; ${N}_{{Combinations}}=6$). **c** Lastly, the correspondence between the unaligned and the aligned principal gradient per subject per session were calculated using the transformation matrices. Source data can be obtained from Supplementary Data 1 and 2.

Identification accuracy using the principal gradient increased substantially and monotonically with the number of gradients used in Procrustes alignment (Fig. 1a). We find that differential identifiability behaves consistently with identification accuracy as long as Fisher’s r-to-z transform is applied (Fig. 1b). In other words, differential identifiability with Fisher’s r-to-z transform applied to within- and between-subject correlations also increases with increasing number of gradients used in alignment.

In contrast, the differential identifiability without Fisher’s r-to-z transform using *r* values decreased with increasing number of gradients used in alignment (see Figs. S2, S6 and S10). We performed this additional analyses to match with the original definition of differential identifiability since differential identifiability originally was computed without Fisher’s r-to-z transform^6^, however, we report these results in the supplementary only, because the lack of equidistance in a metric like Pearson’s correlation could cause differential identifiability to decrease because inconsistent spacing between correlation values may distort the difference between within- and between-subject similarity measures, making it appear as though subjects are less distinguishable, even when their individual patterns become more unique.

For identification accuracy, these findings were consistent across all dimensionality reduction approaches. However, for Laplacian Eigenmaps (“le”), differential identifiability first increases up to 10–15 gradients used in alignment, but then decreases again. Overall, in our robustness analyses, we found that PCA and diffusion map embedding (“dm”) behave quite consistently, whereas Laplacian Eigenmaps yield quite unstable results (Figs. S1–3, S5–7 and S9–11), that often are directly opposing the results for diffusion map embedding and PCA. For instance, when using the Schaefer 400 parcellation, the differential identifiability (with r-to-z transformed correlations) typically increases for PCA and diffusion map embedding across levels of sparsity and different kernel functions, whereas it decreases for Laplacian Eigenmaps. To increase focus in subsequent analyses, we performed gradient extraction using the following recommended parameters: kernel = normalized_angle, sparsity = 0.9, dimensionality reduction technique = diffusion map embedding (“dm”) (see Gradient Extraction and Alignment^22–25,41^).

### Aligned principal gradients take on an increasingly mixed character with increasing number of gradients used in Procrustes alignment

Next, we calculated correspondence between the aligned and unaligned principle gradient per subject per session as the ratio of the maximum to the sum of absolute values in the first column of the Procrustes transformation matrix (see “Methods”—“Procrustes Alignment and Correspondence of Aligned and Unaligned Principal Gradients”; eq. 1). This correspondence indicates the degree to which the first gradient before alignment corresponds to the first gradient after alignment. The correspondence between the aligned gradient and its unaligned version decreased with increasing number of gradients used for alignment (Fig. 1c). This indicates that the principal gradient takes on an increasingly mixed character by assimilating more information from lower gradients.

The decrease in correspondence reflects the increase in the fitting capacity of Procrustes alignment, i.e., accuracy of the alignment, which is a result of higher number of gradients used, essentially resulting in an “overfitted” aligned principal gradient. This is because the alignment can increasingly rely on information from the additional gradients rather than solely using the principal gradient to minimize the difference between the aligned subject-level gradient and the group-level (holdout) template. These findings were consistent in the subsequent robustness analyses varying the parcellations (Schaefer 100, 200 and 400 parcellations). However, results did vary depending on the kernel functions (Spearman, Pearson, normalised angle, Gaussian, cosine, and no kernel). While changes in the kernel function often lead to vertical shifts in overall subject identification or differential identifiability, they did not substantially alter the effect of the number of gradients used in alignment. The biggest changes in the results were induced by changing the dimensionality reduction approaches (diffusion map embedding, principal component analysis, Laplacian eigenmaps) (see supplementary; Figs. S1–S12). In particular, these differences were especially pronounced when using Laplacian Eigenmaps, which appeared more sensitive to the number of gradients used in alignment. In most examples, the direction of the effect was still the same, although there were some notable examples, where the direction of the effect changed: when using normalized_angle or gaussian kernels in combination with Laplacian eigenmaps, identification accuracy first increased drastically when using 3 to 4 gradients in alignment, and then decreased just as drastically again. We also performed these analyses with the minimally processed HCP data to test if the effects could be exacerbated when more motion information is available. However, the results remained reasonably similar (see Supplementary Fig. S13).

To provide a more intuitive understanding of how alignment affects the spatial distribution of gradients, we visualized the cortical patterns of the first and second gradients under different alignment conditions using the Schaefer 400 parcellation. These visualizations, presented at both the group level and for an individual subject with lower typicality of functional connectivity (TFC), illustrate the relative stability of group-average gradient patterns alongside more pronounced variations at the individual level. Detailed cortical maps and corresponding correlation analyses are provided in Supplementary Figs. S13–S17.

### Procrustes alignment correlates with motion signals

**Fig. 2:**
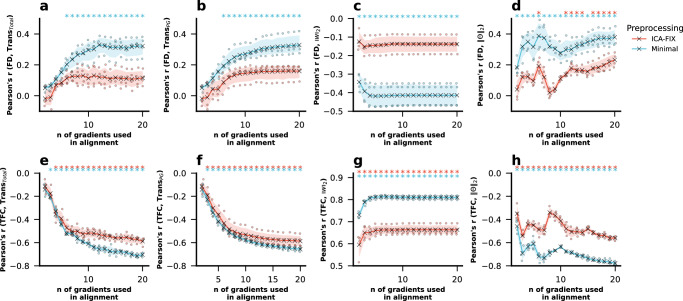
Mean Correlations across the 4 resting state sessions between in-scanner head motion and the magnitude of the transformation performed in Procrustes alignment for varying numbers of gradients used in alignment (fill areas indicate the standard deviation across the 4 resting state sessions). **a** Correlation between FD and the sum of absolute values of all elements in the transformation matrix and **b** correlation between FD and the sum of absolute values of all elements in the first column (i.e., the column that determines the alignment of principal gradient) of the transformation matrix, and **c** the correlation between the ${\ell }_{2}$-norm of the singular values of the Procrustes analysis ${\Vert w\Vert }_{2}$ and FD. **d** Correlation between FD and the ${\ell }_{2}$-norm of the principal angles ($\Vert \varTheta {\Vert }_{2}$). **e** Correlation between TFC and the sum of absolute values of all elements in the transformation matrix, **f** correlation between TFC and the sum of absolute values of all elements in the first column (i.e., the column that determines the aligned principal gradient) of the transformation matrix and **g** the correlation between ${\Vert w\Vert }_{2}$ and TFC, **h** Correlation between FD and the ${\ell }_{2}$-norm of the principal angles ($\Vert \varTheta {\Vert }_{2}$). Asterisks indicate where *p*-values were lower than 0.05 across all resting state sessions after FDR-BH correction for multiple comparisons was applied. Source data can be obtained from Supplementary Data 2, 3, and 4.

One possible source of subject-specific global information is motion in the scanner. We found the average framewise-displacement (FD) was correlated with the magnitude of the transformation (“Trans_Total_”; calculated as the sum of absolute values on the Procrustes transformation matrix; see “Methods”—“Procrustes Alignment and Correspondence of Aligned and Unaligned Principal Gradients”). This correlation increased with an increasing number of gradients used for alignment (Fig. 2a). FD was also correlated with the magnitude of the transformation applied to specifically align the principal gradient (“Trans_PG_”; sum of absolute values of all elements in the first column of the transformation matrix; Fig. 2b). In addition we calculated ${\Vert w\Vert }_{2}$ and $\Vert \varTheta {\Vert }_{2}$ as compact and interpretable descriptors of subspace similarity of the unaligned gradients; see “Methods”—“Procrustes Alignment and Correspondence of Aligned and Unaligned Principal Gradients”). ${\Vert w\Vert }_{2}$ was negatively correlated with FD (high ${\Vert w\Vert }_{2}$ means subspaces are similar) (Fig. 2c) whereas $\Vert \varTheta {\Vert }_{2}$ was positively correlated with FD (high $\Vert \varTheta {\Vert }_{2}$ means subspaces are dissimilar) (Fig. 2d). Importantly, these correlations were stronger and with a steeper increase when using the minimally processed fMRI data (for which motion regression and ICA-FIX were not applied) (Fig. 2a–d), indicating that Procrustes alignment has the capacity to use motion-related information if it is available.

Additionally, TFC—an indicator of motion signal in the subjects’ FC compared to the group FC^36^—was negatively correlated to the total transformation, and this relationship also became more pronounced with increasing number of gradients used in alignment (Fig. 2e). Similar effects are observed for the transformation magnitude specific to the principal gradient (Fig. 2f). Further, ${\Vert w\Vert }_{2}$ was strongly correlated with TFC (Fig. 2g), whereas $\Vert \varTheta {\Vert }_{2}$ was negatively correlated with TFC. Again, these relationships were stronger in fMRI data for which motion regression and ICA-FIX had not been applied. Overall, these findings suggest that the Procrustes transformation captures a higher degree of motion information when more gradients are used in alignment.

We also investigated the degree to which ${\Vert w\Vert }_{2}$ and Trans_Total_ share information by obtaining their correlation for multiple parcellation granularities and numbers of gradients used in alignment (kernel = normalized_angle, approach = diffusion map embedding, sparsity = 0.9) across all datasets (see “Results” in Supplementary Figs. 17–20). Overall, we found moderate Pearson’s *r* correlation coefficients between these two quantities across a multitude of parameters. This suggests that they at least share some of the same information.

To clarify whether the accumulation of motion-related variance is driven specifically by trailing gradients or simply by the inclusion of more gradients in general, we conducted a supplementary analysis addressing this question directly. Specifically, we performed an analysis where gradients were aligned starting from the 20th gradient up to the 1st gradient in a cumulative manner. This reversed alignment approach allowed us to assess whether the accumulation of motion-related effects is specific to trailing gradients or reflects a more general property of including more components in the alignment process. The results of this analysis, showing a gradual increase in correlation with motion metrics as more gradients are included, are presented in Supplementary Fig. S21. This analysis supports the interpretation that the accumulation of motion-related variance is not restricted to trailing gradients but is a general feature of including more gradients in the alignment process.

**Fig. 3:**
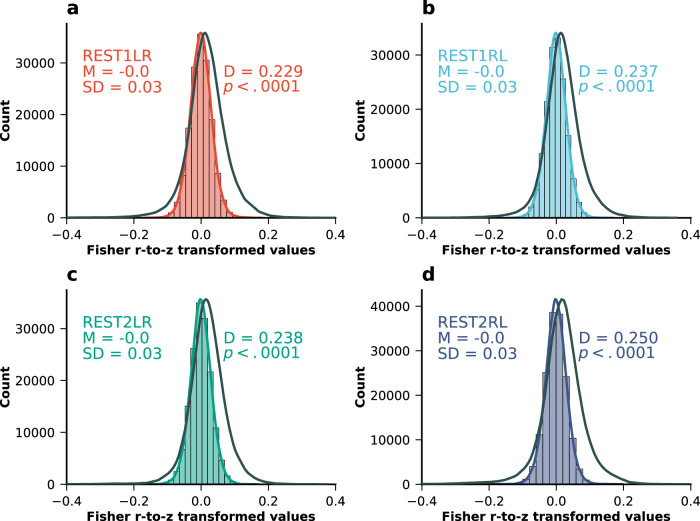
Distribution of correlations between ROI time series and framewise displacement across resting-state sessions. Distribution of correlations between each subjects’ ROI time series and FD time series for all four resting state fMRI sessions in the HCP-YA dataset: **a** REST1LR, **b** REST1RL, **c** REST2LR and **d** REST2RL. The black line corresponds to the distribution of correlation values when using minimally processed data. On the right side of the curves, the *D* test statistic and the associated *p*-value are displayed. These were obtained using a KS test comparing the z-transformed correlation distributions between the minimally processed and the ICA-FIX processed fMRI data. Source data can be obtained from Supplementary Data 5.

To test whether the motion signal present in the transformation matrix of the Procrustes alignment is due to inadequate preprocessing with leftover motion signal, we correlated each subject’s denoised, parcel-wise BOLD time series with the subject’s corresponding FD time series. For each session, this yielded a normal distribution of correlations centered at 0 with a standard deviation of 0.03, indicating that the motion signal was appropriately removed from the BOLD time series (Fig. 3a–d). We additionally compared the distribution of correlations between BOLD and FD time series for ICA-FIX denoised data versus minimally processed data. We applied Fisher’s *r*-to-*z* transformation to all correlation values and conducted Kolmogorov–Smirnov (KS) tests to assess differences in distribution shape. In all four resting-state sessions, we observed that the distribution of correlation values for minimally processed data was significantly different from the distribution for ICA-FIX processed data, with KS statistics ranging from *D* = 0.229 to *D* = 0.250 and all *p* values < 0.0001 (see also Supplementary Fig. S22).

### Procrustes alignment incorporates individual variability in the transformation of the replication data sets

**Fig. 4:**
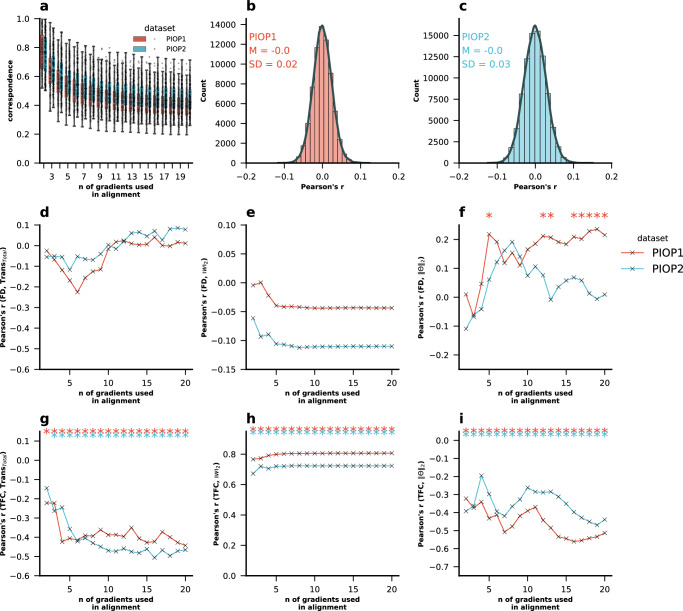
Replication of main analyses in the AOMIC PIOP1 and PIOP2 datasets. Replication of main analyses in the AOMIC PIOP1 and PIOP2 datasets: **a** correspondence of the aligned principal gradient for varying number of gradients used in alignment, **b** Pearson’s correlations between denoised BOLD time series and FD time series in PIOP1 and **c** PIOP2 datasets. **d** Pearson’s correlations between magnitude of the transformation (sum of all absolute values of the transformation matrix) and FD, **e** the correlation between ${\Vert w\Vert }_{2}$ and FD, **f** Pearson’s correlations between FD and $\Vert \varTheta {\Vert }_{2}$ Pearson’s correlations between denoised BOLD time series and FD time series in PIOP1 and **g** Pearson’s correlations between magnitude of the transformation and TFC. **h** The correlation between ${\Vert w\Vert }_{2}$ and TFC. **i** Pearson’s correlations between TFC and $\Vert \varTheta {\Vert }_{2}$. Asterisks indicate where *p*-values were lower than 0.05 after FDR-BH correction for multiple comparisons was applied. Source data can be obtained from Supplementary 2–5.

Next, we sought to replicate key findings regarding the correlation between in-scanner head motion and the magnitude of the transformation performed in Procrustes alignment, particularly in relation to the number of gradients used in alignment. In our analysis of the AOMIC PIOP1 and PIOP2 datasets^42^, again, we found that correspondence between the unaligned and aligned principal gradient decreased with an increasing number of gradients used in alignment (Fig. 4a). We further demonstrate that denoised subject’s ROI-wise BOLD time series do not correlate with motion in the PIOP1 (Fig. 4b) and in the PIOP2 (Fig. 4c) data sets. However, we also did not find the magnitude of the transformation to correlate with FD (Fig. 4d), but we found a strong negative correlation with TFC (Fig. 4g), indicating that Procrustes alignment of gradients leverages individual-level information from additional gradients. Similarly, we did not find significant correlations between ${\Vert w\Vert }_{2}$ and FD (Fig. 4e), but we did find strong correlations between ${\Vert w\Vert }_{2}$ and TFC (Fig. 4h). We observed an increasing correlations between FD and $\Vert \varTheta {\Vert }_{2}$ in the PIOP1 dataset, but not in the PIOP2 dataset (Fig. 4f), but in both datasets, there was a decreasing negative correlations between $\Vert \varTheta {\Vert }_{2}$ and TFC, consistent with the correlations between TFC and TransPG.

**Fig. 5:**
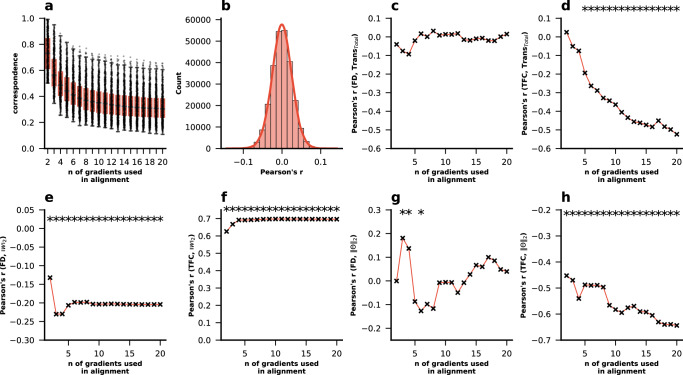
Replication of main analyses in the CamCAN dataset. Replication of main analyses in the CamCAN dataset: **a** correspondence of the aligned principal gradient for varying number of gradients used in alignment, **b** Pearson’s correlations between denoised BOLD time series and FD time series, **c** Pearson’s correlations between magnitude of the transformation (sum of all absolute values of the transformation matrix) and FD, **d** Pearson’s correlations between magnitude of the transformation and TFC, **e** the correlation between ${\Vert w\Vert }_{2}$ and FD, **f** the correlation between ${\Vert w\Vert }_{2}$ and TFC, **g** Pearson’s correlations between TFC and $\Vert \varTheta {\Vert }_{2}$, and **h**) Pearson’s correlations between FD and $\Vert \varTheta {\Vert }_{2}$. Asterisks indicate where *p*-values were lower than 0.05 after FDR-BH correction for multiple comparisons was applied. Source data can be obtained from Supplementary Data 2, 3, 4, and 5.

Since both HCP-YA as well as the AOMIC datasets have a rather narrow age distribution, and since age is related to head-motion in the scanner^43,44^, we sought to replicate our findings in the Cam-CAN dataset, which has a wider age distribution (see “Methods”—Table 1). We found that correspondence again quickly decreased when increasing the number of gradients used in Procrustes alignment (Fig. 5a). We confirmed that the parcellated BOLD time series were not strongly related to FD (Fig. 5b). When extracting gradients and performing Procrustes alignment, the magnitude of the transformation was not strongly related to FD (Fig. 5c), but it was strongly related to TFC (Fig. 5d). However, we did find significant negative correlations between ${\Vert w\Vert }_{2}$ and FD (Fig. 5e), as well as strong positive correlations between ${\Vert w\Vert }_{2}$ and TFC (Fig. 5f). In contrast there was no clear correlation between $\Vert \varTheta {\Vert }_{2}$ and FD (Fig. 5g), but there were decreasing negative correlations between $\Vert \varTheta {\Vert }_{2}$ and TFC (Fig. 5h).

**Table 1:** Age and sex distribution for each of the 4 datasets used, and their respective “analysis” and “holdout” samples

Dataset	Sample	Count	Male	Female	M_Age_	SD_Age_	MIN_Age_	MAX_Age_
**HCP-YA**	**Whole**	**395**	**203**	**192**	**28.74**	**3.82**	**22**	**37**
	Analysis	296	152	144	28.83	3.71	22	37
	Holdout	99	51	48	28.46	4.12	22	35
**AOMIC-PIOP1**	**Whole**	**175**	**78**	**97**	**22.09**	**1.77**	**18**	**26**
	Analysis	131	58	73	22.09	1.81	18	26
	Holdout	44	20	24	22.02	1.66	18	26
**AOMIC-PIOP2**	**Whole**	**212**	**93**	**119**	**21.92**	**1.77**	**18**	**25**
	Analysis	158	70	88	21.81	1.76	18	25
	Holdout	54	23	31	22.25	1.76	19	25
**Cam-CAN**	**Whole**	**627**	**312**	**315**	**54.33**	**18.30**	**18**	**88**
	Analysis	470	234	236	53.80	18.72	18	88
	Holdout	157	78	79	55.92	17.96	18	87

### Procrustes alignment can impact the prediction of fluid intelligence and age

**Fig. 6:**
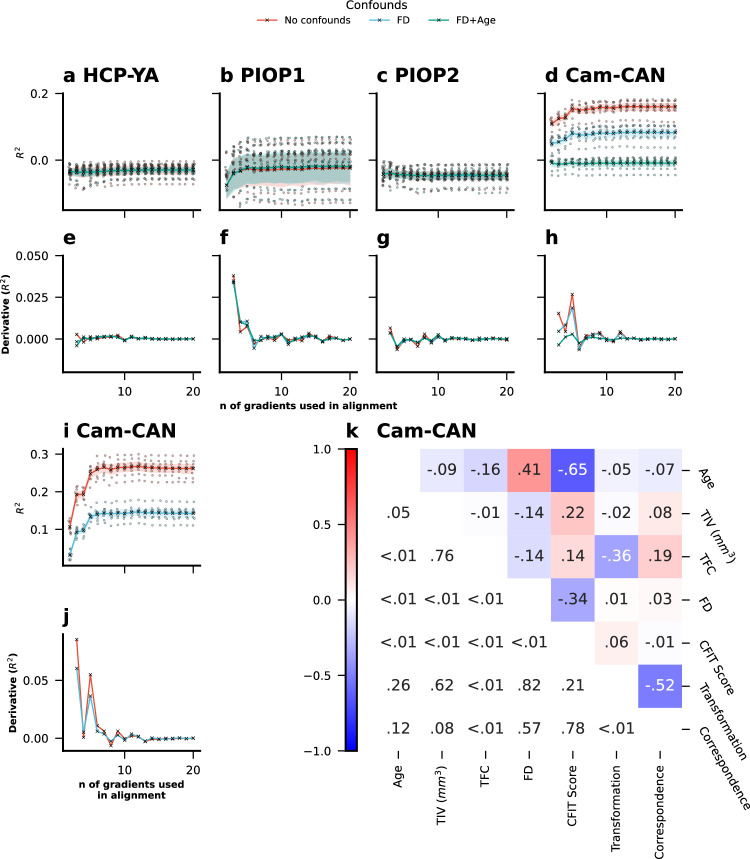
Prediction of cognitive and demographic variables using gradient-based features. Prediction of fluid intelligence using the first principal gradient with varying numbers of gradients used in Procrustes alignment in **a** HCP-YA (PMAT24_A_CR), **b** AOMIC PIOP1 (raven_score), **c** AOMIC PIOP2 (raven_score), and **d** Cam-CAN (CFIT Score) as well as the corresponding derivatives of prediction scores (R^2^) in **e** HCP-YA, **f** AOMIC PIOP1, **g** AOMIC PIOP2, and **h** Cam-CAN datasets. **i** Prediction of age in the Cam-CAN dataset as well as the (**j**) corresponding derivative of prediction scores (R^2^). **k** Correlations between the transformation magnitude, correspondence (when using 10 gradients in Procrustes alignment), CFIT Score, FD, TFC, TIV and Age in the Cam-CAN dataset. The lower left half of the correlation matrix displays the corresponding *p*-values. Source data can be obtained from Supplementary Data 1.

To examine the impact of Procrustes alignment on the prediction of commonly used targets in the FC literature, we sought to predict fluid intelligence, a psychometric measure which is available in all our datasets, using the aligned principal gradient. We could not find clear evidence of prediction in the HCP-YA (Fig. 6a), AOMIC PIOP1 (Fig. 6b), and PIOP2 (Fig. 6c), with R^2^ consistently close to 0. However, we found evidence that the principal gradient can predict fluid intelligence in the Cam-CAN dataset (Fig. 6d) with R^2^ (mean across folds and repeats) reaching a maximum value as high as 0.161 when using 14 gradients in Procrustes alignment. Further, prediction scores decreased drastically when removing FD as a confound, and when using FD and age together as confounds, R^2^ values were close to zero independent of the number of gradients used in alignment. In addition, we calculated the derivative of prediction scores to see the rate of change in prediction scores depending on the number of gradients used in alignment (Fig. 6e–h) and whether this rate of change was different between different confound removal scenarios. However, we did not find clear evidence that confound removal affected this rate of change.

Since this result clearly indicated a relationship between the principal gradient and age, and because age is a common prediction target in neuroimaging research in its own right, we also sought to use the principal gradient to predict age in the Cam-CAN dataset. We found again that increasing the number of gradients used in Procrustes alignment led to higher prediction scores (Fig. 6i) with a mean R^2^ as high as 0.268 when using 12 gradients in Procrustes alignment. Again, we looked at the rate of change, and this time, we did observe that not removing FD as a confound initially led to slightly higher increases of the prediction scores (Fig. 6j). We examined the Cam-CAN dataset, finding strong negative correlations between age and fluid intelligence, a positive correlation between age and motion, and a weak negative correlation between age and TFC, with TFC strongly linked to Procrustes alignment transformation magnitude using 10 gradients (Fig. 6k). In addition, we compared the distributions of average FD values between the four datasets (and the individual HCP-YA sessions), and as expected (due to the wider age range), we find that FD values are overall larger and more dispersed in the Cam-CAN dataset (see Supplementary Fig. S23).

### Prediction of motion signal

**Fig. 7:**
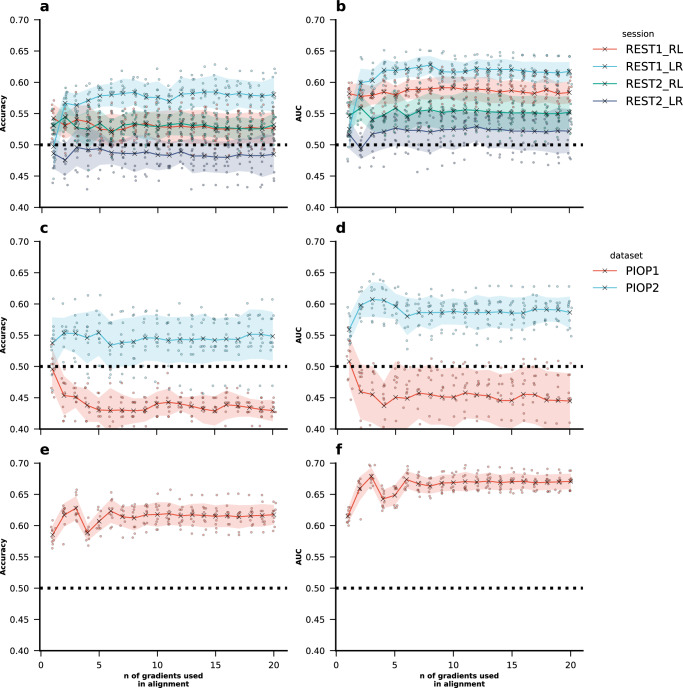
Classification of high- and low-motion subjects using gradient-based features. **a** Classification accuracy (correct predictions divided by total predictions) and **b** area under the receiver operating characteristic curve (AUC) for classification of “high-” and “low-motion” subjects in the HCP-YA, **c** classification accuracy and **d** AUC in the AOMIC PIOP1 and AOMIC PIOP2, and **e** classification accuracy and **f** AUC in the Cam-CAN datasets using ridge classifiers. The dashed black line indicates chance level performance. The shaded area around the lines represents the standard deviation across 10-times repeated fivefold cross-validation. For each repeat performance was averaged across the fivefolds, and then the standard deviation across the 10 resulting mean values was computed. Source data can be obtained from Supplementary Data 1.

To assess the degree to which the number of gradients in Procrustes alignment affects the motion signal present in the principal gradient, we also used a machine learning approach to classify subjects as “high-” or “low-motion” subjects using aligned principal gradients as features. We binarised FD using median FD as a threshold, as we expected a weak signal, and classification tasks are typically easier to solve than regression^45^. Across all four sessions in the HCP-YA dataset, classification accuracy and the area under the receiver operating characteristic curve (AUC) were close to the chance-level, indicating little to no evidence of successful out-of-sample prediction (Fig. 7a, b).

Similar chance-level performance was found in both the AOMIC PIOP1 dataset and the AOMIC PIOP2 dataset (Fig. 7c, d). In the Cam-CAN dataset, with the widest age distribution out of these datasets, we found evidence that classification of high- vs low-motion subjects was above the chance-level (Fig. 7e, f). This result may be attributed to the diverse age distribution in the Cam-CAN dataset, which could introduce additional variability that aids in the classification process. However, in none of the datasets we found a clear pattern of classification accuracy increase or decrease depending on the number of gradients used in Procrustes alignment.

## Discussion

In this study, we investigated the impact of Procrustes alignment on the aligned principal gradient and its implications for subject-level downstream analyses. Specifically, we evaluated the impact of the number of gradients used in alignment. Our findings shed light on several critical aspects regarding the application and interpretation of Procrustes alignment in individual-level functional gradient studies. The results demonstrate that the aligned principal gradient incorporates information not only from the unaligned principal gradient but from all other gradients included in the alignment. We observed a decreasing correspondence between aligned and unaligned principal gradients as more gradients were included. This is likely due to the fact that the current Procrustes alignment treats all gradients included in the transformation equally, regardless of their eigenvalue or the variance explained. A potential alternative approach could consider weighting gradients during alignment, for example, by their eigenvalues, so that the dominant gradients have a proportionally greater influence on the resulting transformation. A similar effect has been reported in the application of Procrustes rotation to PLS permutation testing, and as pointed out by others, it will likely extend to further applications of Procrustes rotation as well^46^.

The number of gradients used in Procrustes alignment strongly influenced identification accuracy and differential identifiability of the principal gradient. As the number of gradients increased, identification accuracy and differential identifiability improved (with Fisher r-t-z transformed correlations). However, as we also point out, differential identifiability without the Fisher r-to-z transform behaves differently and decreases. We point this out, as typically differential identifiability has been computed in the literature without this transformation^6^. This finding may explain why previous research has occasionally found some inconsistencies in the behaviour of identification accuracy and differential identifiability^10,47^. Overall, the impact of the number of gradients used in alignment on identification and identifiability can be explained by the observation that the aligned principal gradient integrates information from all gradients used in the alignment process. This is in particular demonstrated by the decrease of correspondence—the degree to which the first gradient after alignment corresponds to one of the gradients before alignment—when increasing the number of gradients used in alignment.

In addition to the effects of the number of gradients and alignment procedure, the choice of dimensionality reduction method can substantially influence the properties of the resulting gradients. For example, we observed that Laplacian Eigenmaps (LE) often yield low differential identifiability despite relatively high subject identification accuracy. This behavior contrasts with methods such as PCA or Diffusion Map Embedding (DM) and likely reflects fundamental differences in how these algorithms handle variance. LE, as a nonlinear technique, distributes variance more evenly across gradients and emphasizes local connectivity structures, whereas PCA orders gradients by variance explained and DM incorporates a diffusion process that naturally weights eigenvectors by their eigenvalues, emphasizing more global connectivity patterns^20^. These observations suggest that, for applications prioritizing stable principal gradients and cross-subject comparability, nonlinear approaches like LE may be less suitable.

Our results suggest that individual variability (e.g., some motion signals) is captured by the transformation obtained using Procrustes analysis to align the principal gradient. We observed a positive correlation between average FD and the magnitude of Procrustes transformation, which intensified with an increasing number of gradients used for alignment (Fig. 2). This finding is further strengthened by a FC-derived motion measure (TFC) which showed a negative correlation with the magnitude of transformation indicating that lower TFC, associated with higher motion, corresponds to a greater degree of transformation in Procrustes alignment. The correlation between the transformation and TFC was higher than that with FD and was more consistent across datasets. This is expected considering that the purpose of the Procrustes alignment is to make subject-gradients more typical. We would like to note that, although TFC is correlated to measures of motion, it is a measure of how similar a given FC profile is to the group average, and thus it has important limitations as a motion-specific measure. First, TFC is sensitive to *all* deviations from a normative connectivity pattern—including scanner artifacts, respiratory or cardiac noise, preprocessing differences, and even genuine neurobiological variation—rather than isolating head motion effects. Additionally, although TFC captures the overall “quality” of FC (compared to some definition of “normative FC”), it cannot specify *where* or *how much* motion is affecting the data—it offers a broad-strokes estimate rather than targeted diagnostics. Together, these limitations suggest that while TFC is a useful screening tool, it should be complemented with more direct, motion-specific measures when aiming to precisely quantify and correct for head motion.

Finally, we note that the summary metric we used to characterise the strength of the Procrustes transformation, the sum of absolute values of the rotation matrix, has important limitations. In the simple 2 × 2 rotation case, this metric exhibits a non-monotonic relationship with the underlying rotation angle, meaning that transformations that differ substantially from one another can nevertheless produce very similar metric values. For this reason, we view the sum of absolute values of the rotational matrix as a heuristic exploratory descriptor that can illustrate relative changes across conditions. We retain its use because, despite its limitations, the metric consistently captured relative shifts in the magnitude of the estimated transformations across different alignment settings in a way that is stable and interpretable within our analyses. In other words, while it does not quantify absolute alignment distance, it reliably reflects comparative changes across conditions, which is sufficient for the exploratory purposes for which we employed it.

Overall, our results suggest that with more gradients, the Procrustes alignment incorporates a higher degree of individual variability. However, it is an accumulative phenomenon: as more gradients are incorporated—regardless of whether they are leading or trailing—more information is included in the alignment process. It is worth noting that this encompasses both meaningful signals as well as potential nuisance effects such as motion-related artifacts. Importantly, Procrustes alignment does not account for the natural weighting of gradients by their eigenvalues. In the current Procrustes framework, each gradient contributes equally to the alignment, meaning that higher-order gradients can influence the transformation to the same extent as the principal gradient, despite carrying substantially less variance (or “importance”). This lack of weighting provides an additional explanation for why including many gradients can disproportionately alter the principal gradient after alignment. Thus, the inclusion of more gradients introduces both meaningful inter-individual signal and potential nuisance effects not necessarily because trailing gradients are “noisier,” but because their unweighted contribution affects the alignment to the same degree as leading gradients. A potential alternative approach could consider weighting gradients during alignment, for example, by their eigenvalues, so that dominant gradients have a proportionally greater influence on the resulting transformation.

Our machine learning analysis corroborates these findings to some degree. While we did not find evidence of prediction of fluid intelligence using the principal gradient in the HCP-YA and AOMIC datasets, fluid intelligence could be predicted in the Cam-CAN dataset with R^2^ consistently above 0.1. This level of accuracy is plausible given that a FC-based meta-analysis has found Pearson’s r between true and predicted fluid intelligence close to ~0.15^48^. Studies using the Cam-CAN dataset specifically have found prediction scores even above that, likely also due to confounding effects of age, but also possibly due to its use of Cattels’ Culture Fair Intelligence Test (CFIT) rather than Raven’s Progressive Matrices (PMAT) as used in the HCP and AOMIC datasets^49^. More importantly, we found that increasing the number of gradients used in Procrustes alignment increased prediction of fluid intelligence in the Cam-CAN dataset, but that removal of age and motion information (average FD) also removed the principal gradients capacity to predict fluid intelligence independent of the number of gradients used in alignment. Similarly, we found that we could successfully predict age using the principal gradient in the Cam-CAN dataset, and that increasing the number of gradients used in Procrustes alignment also increased prediction scores. Importantly, when removing the confounding effect of average FD, prediction scores dropped, and in addition, the increase in prediction scores with each additional gradient used in alignment decreased slightly. This indicates that in-scanner head motion contributes to better prediction success when more gradients are used in alignment.

Lastly, we attempted to predict the subject-level head motion using the principal gradient. We expected better prediction success with more gradients used in Procrustes alignment. While we did not find clear evidence of successful classification of motion in the HCP-YA and AOMIC datasets, we found good evidence of successful classification in the Cam-CAN dataset. This may be a result of the relatively low sample sizes in the former datasets, which is not favourable for machine learning analysis. In contrast, the Cam-CAN dataset has the largest sample size as well as more variance of age and FD. This is important since age is related to head-motion in the scanner^43,44^ as well as gradients^23^ and a wider age range is therefore likely to improve gradient-based prediction of motion. However, even in the Cam-CAN dataset we did not find evidence for increased classification accuracy with more gradients used in Procrustes alignment.

One limitation affecting our ability to predict motion in the classification analysis is the inherent dilution of the motion signal accumulated over multiple stages of analysis. FD, commonly used as a measure of motion, is derived indirectly from the fMRI time series, where each time point reflects a snapshot of head position changes across translational and rotational dimensions. As a result, FD captures movement indirectly rather than directly reflecting physiological or anatomical features related to motion. Furthermore, by averaging FD values across all time points for each subject, the dynamic variability of motion over the scanning session is reduced to a single summary statistic, which can mask significant temporal fluctuations and lead to further signal loss. These factors mean that the motion signal is both indirect and reduced, limiting the ability of our classification models to accurately classify subjects based on their average FD.

In addition, although FD provides a useful proxy for in-scanner head motion, it captures only a partial aspect of motion-related artefacts^50^. Importantly, FD is a catch-all measure, and similar FD values can have multiple underlying types of motion^51^. Moving forward, future studies could benefit from incorporating more direct measures of motion using external cameras or motion-tracking devices^52,53^. These technologies can provide real-time, high-resolution data on head movements, allowing for a more comprehensive understanding of motion artefacts during MRI scans.

These results have significant implications for the interpretation and application of Procrustes alignment in functional gradient studies, especially for individual-level analysis. The observed relationship between alignment characteristics and motion signals underscores the importance of considering motion artefacts in FC analyses, particularly in individual-level studies where motion can significantly impact data quality and interpretation. For example, it has been found that motion signals reliably correlate with behavioural outcomes^39,54^. While motion signals should be primarily addressed in denoising prior to computing correlation matrices, motion signals can persist in FC data even after extensive processing independent of the specific denoising pipeline chosen^35–39^. Moreover, aggressive preprocessing can reduce artifacts, but it can also remove substantial amounts of variance of interest and potentially diminish meaningful intersubject variability, highlighting the trade-off between cleaning fMRI data and preserving biologically relevant individual differences^55^. Additionally, some strategies to account for motion (e.g., excluding high-motion subjects) can bias datasets and lead to understudying of important clinical populations^56,57^. Therefore, rather than excluding subjects with high head-motion, it is often suggested to employ appropriate strategies to address motion artefacts, ensuring that analyses remain robust and inclusive^38,58,59^. To this end, it may also be worthwhile exploring the prospect of alternative gradient alignment solutions.

Moreover, the findings emphasise the need for careful consideration of the number of gradients used in alignment, as it directly influences the characteristics and interpretability of the aligned principal gradient. While using a higher number of gradients can increase the fit between group- and individual-level gradients^28,29^, this comes as a trade-off: Specifically, the benefit in fit may not stem from properties of the trailing gradients per se, but from the accumulation of additional variance. Because the alignment procedure treats all gradients equally, including higher-order gradients, affects the transformation of the principal gradient, which can complicate interpretation of its original individual-level properties. Intuitively, aligning with a high number of gradients may be thought of as a kind of “overfitting”. That is, alignment can use the additional variance to transform the principal gradient, making it difficult to make conclusions about individual-level properties of the original principal gradient. As a result, it may not be desirable to recommend a specific number of gradients to be used in alignment, but to consider the advantages of a better group-level fit and the disadvantages it may incur in terms of the inflated importance of the trailing gradients, since they are weighted the same as the principal gradients.

To some degree, our results showcase one of the reasons for extracting gradients in the first place: identify gradients that are of interest for the analysis at hand, and discard those that are not^13^. It may thus be more beneficial to make the choice of number of gradients to align based on specific biological models^15,17,30,60^ rather than using an ad-hoc predefined number^28,29^. Researchers can also perform robustness analyses that test whether their results hold for different numbers of gradients used in Procrustes alignment. These robustness checks can be informative whether the impact of alignment on the results is practically meaningful. In addition, researchers should report the exact number of gradients used in Procrustes alignment, and ideally report whether they did or did not try their analysis with varying numbers of gradients. Additionally, it is important to consider head motion as a confound in studies of functional gradients, and determining the most effective approach to address this remains an active area of research^58^. On the other hand, additional gradients may contain meaningful signals relevant to certain research studies. In such cases, it is reasonable to both extract and align with the full set of gradients intended for analysis.

In conclusion, our study provides valuable insights into the impact of Procrustes alignment on individual-level functional gradient analyses, highlighting its role in integrating information from multiple gradients. While including more gradients can improve group-level fit, the equal weighting of all included gradients can disproportionately inflate the influence of lower-variance gradients. Future work could explore weighting schemes to prioritize the most informative gradients while retaining the benefits of multi-gradient alignment.

## Methods

### Datasets

For this project, we used 4 different datasets: the Human Connectome Project—Young Adults (HCP-YA) dataset, the AOMIC Population Imaging of Psychology (PIOP) datasets 1 and 2, and lastly the Cam-CAN dataset. Each dataset was split into two partitions: A holdout sample consisting of 20% of the data was used to create a group average gradient that could be used as a reference for Procrustes alignment, whereas 80% of the data was used to create individual-level gradients for subject-level analyses. The rationale for this choice was to prevent data leakage during cross-validation (CV) in the machine learning pipelines if all participants’ data were used to derive the reference gradients. Age, sex, as well as the partitioning of the data can be seen in Table 1. Retrospective analysis of these datasets was further approved by the local Ethics Committee at the Faculty of Medicine at Heinrich-Heine-University in Düsseldorf (2018-317-RetroDEuA). For all datasets used in this study, informed consent was obtained by the original study investigators in accordance with local ethics committee approvals and the Declaration of Helsinki. All ethical regulations relevant to human research participants were followed.

### Human connectome project—young adult

For the primary, exploratory analyses in this project, we used the HCP-YA dataset. The details regarding collection of behavioural data, fMRI acquisition, and image preprocessing in the HCP-YA have been described elsewhere^40,61,62^. Here, we aim to give a brief overview. The scanning protocol for the HCP-YA was approved by the local Institutional Review Board at Washington University in St. Louis. We used data obtained from four resting-state fMRI (rs-fMRI) sessions.

The four sessions of rs-fMRI were obtained on two separate days (each lasted ca. 15 min; ~60 min across all four sessions). On each day, two sessions were recorded for different phase encoding directions (left-right [LR] and right-left [RL]), providing four overall rs-fMRI datasets. Scans were acquired using a 3 T Siemens connectome-Skyra scanner with a gradient-echo EPI sequence (TE = 33.1 ms, TR = 720 ms, flip angle = 52°, 2.0 mm isotropic voxels, 72 slices, multiband factor of 8). We used volumetric data, and this data had already undergone the HCP’s ICA-FIX (independent component analysis and FMRIB’s ICA-based X-noiseifier) procedure^63^, which also included removal of Friston 24 motion parameters^62^. In addition, we regressed out white matter (WM), cerebro-spinal fluid (CSF), and global signals (GS), their squared terms, and temporal derivatives. The data was bandpass filtered at 0.01–0.08 Hz.

### Amsterdam open MRI collection

We used two datasets from the AOMIC, known as the PIOP data sets 1 and 2^42^ to replicate and validate our main findings. The exact data collection protocols are described elsewhere in detail, so here we provide a short overview. The University of Amsterdam’s ethical committee approved these studies before data collection started (PIOP1 EC number: 2015-EXT-4366, PIOP2 EC number: 2017-EXT-7568).

The functional MRI scanning protocol for the PIOP1 and PIOP2 data sets utilised a Philips 3 T scanner, with PIOP1 scanned on the “Achieva” version and PIOP2 on the “Achieva dStream” version, both equipped with a 32-channel head coil. Functional MRI data in PIOP1 for resting-state fMRI were acquired with multiband acceleration, whereas functional MRI scans in the PIOP2 were acquired sequentially. For both data sets, resting state scans lasted ~6 min (PIOP1; i.e., 480 volumes with a 0.75 s TR) and ~8 min (PIOP2; i.e., 240 volumes with a 2 s TR).

We used volumetric data processed using fMRIPrep as provided by the original PIOP studies^42,64^. Spatial normalisation to the ICBM 152 Nonlinear Asymmetrical template was performed using nonlinear registration with ANTs. For functional data, motion correction was conducted using mcflirt, and “fieldmap-less” distortion correction was applied by co-registering functional images to T1-weighted images with intensity inversion. Similar to the HCP-YA dataset, we regressed out the Friston 24 motion parameters, WM, CSF and GS, their squared terms, and temporal derivatives. The data was bandpass filtered at 0.01–0.08 Hz.

### Cambridge centre for ageing and neuroscience

Because both HCP-YA and the AOMIC datasets have a rather narrow age distribution with mainly young adults, we also sought to replicate our findings using resting state fMRI data from the Cam-CAN dataset. Collection of this data has been approved by the Cambridgeshire 2 Research Ethics Committee (ref. ^10^:/H0308/50). Data from Cambridge Centre for Ageing Neuroscience dataset were retrieved from their official source at the CamCAN Data Portal (https://camcan-archive.mrc-cbu.cam.ac.uk/dataaccess/)^65,66^ and provided in the form of a DataLad dataset, a research data management solution providing data versioning, data transport, and provenance capture^67^.

Resting-state fMRI data in the Cam-CAN dataset were acquired using a T2*-weighted Gradient-Echo Echo-Planar Imaging (EPI) sequence while participants rested with their eyes closed. A total of 261 volumes were collected, each comprising 32 axial slices acquired in descending order. The slices had a thickness of 3.7 mm with an interslice gap of 20%, ensuring whole-brain coverage including the cerebellum. The acquisition parameters included a repetition time (TR) of 1970 milliseconds, an echo time (TE) of 30 milliseconds, a flip angle of 78 degrees, and a field of view of 192 × 192 mm. The voxel size was 3 × 3 × 4.44 mm, and the total acquisition time for each scan was 8 min and 40 s.

In order to keep preprocessing as consistent as possible, we processed the volumetric data using fMRIPrep with similar configurations to the AOMIC datasets. Spatial normalisation to the ICBM 152 Nonlinear Asymmetrical template was performed using nonlinear registration with ANTs. For functional data, motion correction was conducted using mcflirt. Again, we regressed out the Friston 24 motion parameters, WM, CSF, and GS, their squared terms, and temporal derivatives. The data was bandpass filtered at 0.01–0.08 Hz.

### Gradient extraction and alignment

Denoised time series were aggregated using the Schaefer 400 parcellation^68^, a popular choice for FC-based prediction and functional gradient studies^9,22,28^. FC was calculated as pairwise Pearson’s correlation between ROI time series resulting in a 400 × 400 connectivity matrix per subject per session. For subjects in the main analysis dataset, gradients for each session were extracted from these FC matrices using the BrainSpace toolbox^16^, which also provides Procrustes alignment to find the optimal transformation matrix to minimise the sum of squared errors of a source matrix to a reference matrix. The BrainSpace toolbox provides 5 different kernel functions and 3 different dimensionality reduction techniques. A kernel function computes the relationship between each pair of parcels and derives a non-negative square symmetric affinity matrix ${{\bf{A}}}$, where each element ${{\bf{A}}}(i,j)$ describes the affinity between parcels i and j. Brainspace provides implementations for the following kernel functions:

1. Gaussian kernel
  $${{\bf{A}}}(i,j)={e}^{-(\gamma \Vert x-y{\Vert }^{2})}$$
  ${{\bf{A}}}(i,j)={e}^{-(\gamma \Vert x-y{\Vert }^{2})}$ where *γ* is the inverse kernel width and ||■|| denotes the ${\ell }_{2}$ -norm.
2. Cosine similarity
  $${{\bf{A}}}(i,j)=cossim({{\bf{x}}},{{\bf{y}}})=\frac{{{\bf{x}}}{{{\bf{y}}}}^{T}}{\Vert {{\bf{x}}}\Vert \Vert {{\bf{y}}}\Vert }$$
  where *cossim* is the cosine similarity function and *T* stands for transpose.
3. Normalized angle similarity
  $${{\bf{A}}}(i,j)=1-\frac{{\cos }^{-1}(cossim({{\bf{x}}},{{\bf{y}}}))}{\pi }$$
4. Pearson correlation
  $${{\bf{A}}}(i,j)=\rho ({{\bf{x}}},{{\bf{y}}})=cossim({{\bf{x}}}-\overline{{{\bf{x}}}},{{\bf{y}}}-\overline{{{\bf{y}}}})$$
  where ***ρ*** is the Pearson correlation coefficient, and $\overline{{{\bf{x}}}}$ and $\overline{{{\bf{y}}}}$ denote the means of x and y, respectively.
5. Spearman rank order correlation

$${{\bf{A}}}(i,j)=\rho ({{{\bf{r}}}}_{{{\bf{x}}}},{{{\bf{r}}}}_{{{\bf{y}}}})$$

where ***r***_*x*_ and ***r***_*y*_ denote the ranks of ***x*** and ***y***, respectively.

The dimensionality reduction techniques are then used to find a low-dimensional representation. BrainsSpace provides implementations for diffusion map embedding (“dm”), principal component analysis (“pca”), and Laplacian Eigenmaps (“le”). For more detail on how these work, we refer the reader to the excellent paper outlining the implementation of these techniques in the BrainSpace toolbox^16^.

Here, we mostly focus on gradient extraction using the following recommended parameters: kernel = normalized_angle, sparsity = 0.9, dimensionality reduction technique = diffusion map embedding (“dm”). These parameters are among the most commonly used in the literature^15,22–25,41^. In addition, as a robustness check, we provide results for alternative parameters in the supplement.

For subjects in the 20% holdout dataset, FC matrices were averaged after applying Fisher’s r-to-z transform. Elements in this average matrix underwent the reverse transform to derive correlation values and using this average FC matrix, an average reference gradient set was derived for alignment. The gradients of subjects in the analysis dataset were then each aligned to this holdout reference gradient using Procrustes alignment. Subsequent downstream analyses were performed using these aligned gradients. The reference was derived separately for each of the four datasets.

### Procrustes alignment and correspondence of aligned and unaligned principal gradients

Before comparing gradients, it is important to align them for two main reasons: Firstly, the subject-level and group-level gradients can be in a different order, e.g., the second subject-level gradient might match better with the reference principal gradient. Secondly, the sign of parcels within gradients can also vary arbitrarily across subjects. Procrustes alignment aims to solve this by linearly transforming a given set of unaligned gradients to optimally align with a set of reference gradients. For example, let ${{\bf{A}}}$ be a $m\times n$ matrix containing a subject’s $n$ gradients consisting of $m$ ROIs, and ${{\bf{B}}}$ be the equivalent $m\times n$ reference gradient matrix. Procrustes alignment is then performed using an $n\times {n}$ transformation matrix ${{\bf{T}}}$ to transform ${{\bf{A}}}$ such that the sum of squared errors between ${{\bf{B}}}$ and the transformed matrix $A{\prime}$ is minimised, i.e., $A{\prime} =AT$.

$$T=(\begin{array}{ccccccc}{t}_{11} & \cdots & {t}_{1n}\vdots & \ddots & \vdots {t}_{n1} & \cdots & {t}_{nn}\end{array})$$

The transformation matrix ${{\bf{T}}}$ is obtained by employing generalized Procrustes analysis, a method that iteratively finds the best orthogonal transformations to minimize the pairwise distances between the aforementioned sets of unaligned gradients A and reference gradients B. The transformation is computed using SVD of the cross-covariance matrix C:

$${{\bf{C}}}={({{{\bf{B}}}}^{\top }{{\bf{A}}})}^{\top }$$

$${{\bf{U}}}{{\boldsymbol{\Sigma }}}{{{\bf{V}}}}^{\top }={{\rm{SVD}}}({{\bf{C}}})$$

$${{\bf{T}}}={{\bf{U}}}{{{\bf{V}}}}^{\top }$$

Intuitively, the cross-covariance matrix captures how the gradients of one dataset relate to those of the reference. SVD decomposes this relationship into a series of rotations and scalings that best explain how one dataset can be reoriented to match the other. In the context of Procrustes alignment, this decomposition provides the most efficient way to rotate one set of gradients so that it aligns as closely as possible with the reference set. This approach was formally described and solved by ref. ^69^. who showed that the SVD-based method provides a closed-form solution to the orthogonal Procrustes problem—i.e., finding the optimal rotation to align two sets of points in a least-squares sense.

Importantly, we did not use generalised Procrustes alignment. We decided against this because in generalised Procrustes alignment, the reference is iteratively updated using information from all subjects, which introduces data leakage across subjects included in the analysis. This compromises the independence of train-test splits in subsequent machine learning analyses, violating a key requirement for proper model evaluation and generalization. Instead, we chose to align each subject individually to a fixed reference using standard (pairwise) Procrustes alignment, thereby preserving subject independence.

Since the aligned principal gradient is a linear combination of the original, unaligned gradients, the transformation matrix provides information about each unaligned gradients’ contribution toward the aligned principal gradients. To quantify this, we calculated the relative magnitude of the unaligned principal gradient with respect to all gradients used for alignment. If the contribution of the unaligned principal gradient toward the aligned principal gradient is relatively large, then we can conclude that the unaligned and the aligned principal gradient have a high correspondence with each other.

This “Correspondence” is calculated as the ratio of the maximum to the sum of absolute values in the first column of the transformation matrix ${{\bf{T}}}$, which contains the weights for each original unaligned gradient toward the aligned principal gradient. That is, ${t}_{11}$ represents the contribution of the first unaligned gradient toward the aligned principal gradient, and more generally, ${t}_{i1}$ represents the contribution of the $i$-th unaligned gradient toward the aligned principal gradient. Thus, the values in the first column of ${{\bf{T}}}$ can be interpreted in relation to each other. Thus, the maximum value (irrespective of the sign) represents the unaligned gradient that contributed the most to the aligned principal gradient, while all other unaligned gradients contribute correspondingly to the aligned principal gradients. Thus, the “correspondence” measure quantifies the degree to which the most contributing unaligned gradient is represented in the aligned principal gradient.

More formally, to quantify the correspondence between the unaligned and aligned principal gradients (i.e., the first column of the untransformed original matrix ${{\bf{A}}}$ and the transformed matrix $A{\prime}$), we calculate the correspondence as follows:

$$c=\frac{\max ({{\vert}}{t}_{,1}{{\vert}})}{{\sum }_{i=1}^{n}{{\vert}}{t}_{i1}{{\vert}}}$$

Here $|{t}_{i1}|$ represents the absolute value of each element. This expression accounts for possible reordering of the unaligned gradients as well as change in the sign.

In other words, $c$ quantifies how dominant the largest weight is compared to the overall weights in the first column of the transformation matrix ${{\bf{T}}}$. It quantifies how dominantly the unaligned principal gradient determines the aligned principal gradient compared to all other gradients. That is, if $c$ is close to 1, it suggests that the transformation is dominated by the unaligned principal gradient, indicating its strong correspondence with the aligned principal gradient. Conversely, if $c$ is closer to 0, it implies a more distributed adjustment using all unaligned gradients, indicating a weaker correspondence.

We also calculated the magnitude of the Procrustes rotation as the sum of absolute values in the transformation matrix, to get a sense of the degree to which the rotation conveys information on subject motion. This sum provides a quantitative measure of the extent of alignment required to match the subject data to the reference. To further dissect the impact of Procrustes rotation, we specifically focused on the alignment of the principal gradient - a key component in functional gradient analysis. For this, we calculated the magnitude of the transformation applied to align the principal gradient as the sum of absolute values in the first column of the transformation matrix. This column determines the transformation of the principal gradient during Procrustes alignment, and the magnitude of its summed elements can thus indicate how much the principal gradient is altered to achieve alignment. A higher sum would suggest a greater transformation, implying that the subject’s brain data diverged more from the reference, possibly due to subject motion or intrinsic variability.

In addition to examining the transformation matrix directly, we also consider the singular values $w$ obtained during the SVD step of the Procrustes alignment. These singular values quantify how well the subspaces spanned by the original (unaligned) and reference gradients correspond to each other. To obtain a compact and interpretable descriptor of subspace similarity, we calculate the ${\ell }_{2}$-norm of the singular values, ${\Vert w\Vert }_{2}$.

We further characterize subspace similarity by examining the principal angles obtained between the original (unaligned) and reference gradient subspaces. These angles provide a direct geometric measure of how much the subspaces deviate from one another. To summarize this information in a single, interpretable quantity, we compute the ℓ₂-norm of the principal angle vector, $\Vert \varTheta {\Vert }_{2}$. This measure captures the overall magnitude of subspace misalignment: smaller values indicate that the subspaces are more closely aligned, whereas larger values reflect greater divergence across the gradient dimensions. Importantly, this measure is sensitive to the combined alignment across all gradient dimensions, and unlike the sum of absolute transformation weights, it reflects the actual geometric agreement between the subspaces of the unaligned matrices. This measure reflects the actual geometric agreement between the subspaces of the unaligned matrices. Importantly, the ${\ell }_{2}$ -norm of principal angles $\Vert \varTheta {\Vert }_{2}$ and the ${\ell }_{2}$ -norm of singular values of the Procrustes transformation ${\ell }_{2}$ act as opposing indicators of subspace similarity (if the subspaces are similar $\Vert \varTheta {\Vert }_{2}$ is low and ${\Vert w\Vert }_{2}$ is high; if the subspaces are not similar $\Vert \varTheta {\Vert }_{2}$ is high and is ${\Vert w\Vert }_{2}$ low).

### Identification and differential identifiability

We performed identification^8^ and calculated differential identifiability^6^ to test the impact of varying the number of gradients used in Procrustes alignment on downstream subject-level analyses. Identification was performed by utilising an individual’s principal functional gradient derived from a resting state fMRI scanning session to find the best match from a database of principal functional gradients derived from another resting state fMRI scanning session. The identification accuracy is determined by the proportion of participants correctly identified. Differential identifiability is determined in a similar fashion by correlating each subject’s principal functional gradient derived in one fMRI scanning session with each subject’s principal gradient derived in another fMRI scanning session. Differential Identifiability between those two sessions is then defined as the difference between mean within-subject correlations (${I}_{{self}}$) and mean between-subject correlations (${I}_{{others}}$): ${I}_{{Diff}}=({I}_{{self}}-\,{I}_{{other}})\times 100$. However, simply subtracting ${I}_{{other}}$ from ${I}_{{self}}$ may be problematic as Pearson’s r is not a distance metric, because it does not measure distance in a way that would allow for straightforward comparison or summation. For instance, Pearson’s *r* is not strictly equidistant, meaning that the “distance” between two points in the metric space does not consistently reflect their relative closeness. In other words, it may fail to maintain equal spacing between pairs of points that intuitively would be considered equally distant^70^. Therefore, we performed an additional analysis in which Fisher’s r-to-z transform is first applied to all correlation values before computing the difference. Identification accuracy and differential identifiability were calculated for each pairwise combination of sessions in the HCP-YA dataset (${N}_{{Sessions}}=\,4$; ${N}_{{Combinations}}=6$).

### Motion signal

#### Framewise displacement

Motion was characterised using average framewise displacement (FD) for each subject. FD is a composite measure derived from six head motion parameters, encompassing translational displacements along the X, Y and Z axes, as well as rotational displacements of pitch, yaw, and roll, transitioning from one volume to the subsequent^37^. It represents the mean disparity among rotation and translation parameters. FD was calculated for each volume in the fMRI data and then averaged to obtain an aggregate measure of motion for the session.

#### Typicality of functional connectivity

In addition to examining motion through FD, we incorporated the concept of TFC as an additional measure of motion derived directly from FC, which has been shown to negatively correlate with measures of head motion in the scanner. While FD captures motion within a subject based on differences between volumes, TFC offers a complementary perspective by assessing motion across subjects relative to the group mean^36^. In addition, FD is derived from the raw time series, whereas TFC serves as a marker of motion of the FC directly, after all processing and denoising has taken place. TFC is defined as:

$$TF{C}_{i}=\frac{1+{r}_{p}(F{C}_{i},\overline{FC})}{2}$$

where ${r}_{p}$ is Pearson’s correlation, ${{FC}}_{i}$ a subject’s FC, and $\overline{FC}$ is the group average (i.e., typical FC). In this study, this group average $\overline{FC}$ was derived from the 20% holdout (see Table 1).

### Prediction of fluid intelligence and age

To investigate the impact of Procrustes alignment on common machine learning workflows, we attempted to predict fluid intelligence across all datasets, and age specifically in the Cam-CAN dataset due to its diverse age range (see Table 1). As a primary model, we used a ridge regression as implemented by scikit-learn^71^. To estimate the generalisation error of the predictive models, we employed 10 times repeated fivefold nested CV. Within each nested CV, an inner fivefold CV was used on each train partition of the respective splits to determine the optimal hyperparameters. For the ridge classifier, we tuned the “alpha” parameter using a grid generated by numpy^72^ as “np.geomspace(0.001, 10000, 50)”. In the HCP-YA and AOMIC datasets, we used Raven’s Progressive Matrices (PMAT24_A_CR in the HCP-YA and raven_score in the AOMIC datasets). In the Cam-CAN dataset, we used Cattel’s CFIT Total Score. In the prediction of fluid intelligence, we followed three confound removal scenarios: (1) Remove no confounds, (2) remove FD as a confound, and (3) remove FD and age as a confound. In the prediction of age, we followed two confound removal scenarios: (1) Remove no confounds, and (2) remove FD as a confound. Each time confounds were removed in a CV consistent way, i.e., by training the confound removal model on the train partition only, and then applying it to the test partition as suggested in previous literature^9,10,73,74^. FD and age were in particular considered as confounding variables, because they have been found to be both associated to FC as well as behavioural and psychometric measures^9,10,39^.

### Prediction of motion

To investigate the individual-level motion-related information present in the principal gradient after Procrustes alignment, we used machine learning to predict the subjects’ head-motion using the aligned principal gradient as features. To this end, we binarized FD scores to create unambiguous classification outcomes using median FD as threshold, such that subjects with below median FD were considered “low-motion” subjects, whereas subjects with above median FD were considered “high-motion” subjects. This choice was motivated by the expectation of a weak effect and the practical advantages of classification in detecting subtle signals. Our goal in this analysis was to investigate whether any effect exists at all between Procrustes alignment and head motion, rather than to precisely estimate motion values. Binary classification, in contrast to regression, is a simpler problem and more robust in detecting weak but systematic patterns. A similar binarization approach has been proposed previously^45^. As a primary model, we used a ridge classifier as implemented by scikit-learn^71^. In addition, we performed this analysis using a linear support vector machine (SVM) as well as SVMs with a radial basis function (RBF) kernel as well as random forest (RF) classifiers to reproduce the results using non-linear models.

To estimate the generalisation error of the predictive models, we employed 10 times repeated fivefold nested CV. Within each nested CV, an inner fivefold CV was used on each train partition of the respective splits to determine the optimal hyperparameters. For the ridge classifier, we tuned the “alpha” parameter using a grid generated by numpy^72^ as “np.geomspace(0.001, 10000, 50)”. For the linear SVM, we generated a grid to optimise the C parameter (“np.geomspace(1e-4, 1e4, 10)”). In the RBF SVM, we additionally optimised the $\gamma$ parameter of the RBF kernel (“np.geomspace(1e-9, 1e4, 10)”). For the RF classifier, we tuned the “max_depth” parameter using the grid “[5, 10, 20, None]”. After hyperparameter selection a model was fitted on the whole train partition and then tested on the left-out test samples of the CV split.

Classification accuracy was computed as the proportion of correctly predicted labels on the test set:

$${{\rm{Accuracy}}}=\frac{1}{N}\mathop{\sum }_{i=1}^{N}({y}_{i}={\hat{y}}_{i})$$

where $(N)$ is the number of test samples, $({y}_{i})$ is the true label, $({\hat{y}}_{i})$ is the predicted label for sample $(i)$.

### Statistics and reproducibility

All analyses were conducted using previously collected, publicly available datasets, and no statistical methods were used to predetermine sample size. Sample sizes were therefore fixed by data availability and are comparable to or larger than those used in prior studies employing FC gradients and Procrustes alignment. Analyses were performed across four independent datasets: the Human Connectome Project Young Adult (HCP-YA), AOMIC PIOP1 and PIOP2 (AOMIC), and the Cam-CAN. For each dataset, participants were split into a holdout sample (20%) used exclusively to derive group-level reference gradients and an analysis sample (80%) used for all subject-level analyses, in order to avoid data leakage in downstream analyses. Exact sample sizes, sex distributions, and age ranges for each dataset and partition are reported in Table 1. All reported analyses were performed independently within each dataset using identical pipelines wherever possible.

Subject identification and differential identifiability analyses were conducted using principal functional gradients derived from 4 resting-state fMRI sessions in the HCP-YA dataset using all subjects included in the main analysis sample (*n* = 296; see Table 1 for sample characteristics). Identification accuracy was defined as the proportion of subjects correctly matched across sessions based on maximum Pearson correlation of the aligned principal gradient. Differential identifiability was computed as the difference between mean within-subject and between-subject correlations. Because Pearson’s correlation coefficient is not a true distance metric, analyses were performed both with and without Fisher’s *r*-to-*z* transformation; results based on *z*-transformed correlations are reported in the main text, while non-transformed results are provided in the Supplementary Material.

In a follow-up analysis, we quantified the relationship between subject-level motion metrics and the magnitude of Procrustes alignment as a function of the number of gradients used for alignment. Analyses were performed in the HCP-YA dataset using all subjects included in the main analysis sample (*n* = 296; see Table 1 for sample characteristics). Framewise displacement (FD) and TFC were computed for each subject and session, and correlations with Procrustes transformation metrics (total transformation magnitude, transformation magnitude specific to the principal gradient, and subspace similarity measures; see Fig. 2) were assessed using Pearson’s correlation coefficient. Correlation coefficients were computed independently for each number of gradients used in Procrustes alignment. Statistical inference relied on two-tailed tests of correlation coefficients, and no assumptions of normality beyond those inherent to Pearson correlation were made. To account for multiple comparisons across sessions, *p* values were corrected using the Benjamini–Hochberg false discovery rate (FDR) procedure with a significance threshold of *q* < 0.05. Correction was applied separately for each preprocessing pipeline and each motion metric. A given alignment dimensionality was considered significant only if all session-specific correlations survived FDR correction. Significant effects are indicated by asterisks in Fig. 2. Results were reproduced across multiple resting-state sessions within HCP-YA and replicated in three independent datasets (AOMIC PIOP1, *n* = 131; AOMIC PIOP2, *n* = 158; Cam-CAN, *n* = 470; see Figs. 4 and 5), demonstrating robustness to differences in acquisition protocols, preprocessing pipelines, and age distributions. All analyses were deterministic and reproducible given fixed preprocessing and alignment parameters.

Figure 3 assesses residual motion-related signal in denoised BOLD time series by examining the distribution of parcel-wise correlations between BOLD time series and framewise displacement (FD) in the complete HCP-YA dataset (analysis and holdout, *n* = 395). For each resting-state session (REST1LR, REST1RL, REST2LR, REST2RL), Pearson’s correlation coefficient was computed between the FD time series and each parcel’s denoised BOLD time series, yielding a distribution of correlation coefficients per session. To formally test whether residual motion effects differed between preprocessing pipelines, distributions obtained from ICA-FIX denoised data were compared to those from minimally processed data using two-sample Kolmogorov–Smirnov (KS) tests. All correlation values were Fisher *r*-to-*z* transformed prior to statistical testing. Across all four sessions, KS tests revealed significant differences between preprocessing pipelines (REST1LR: *D* = 0.229, *p* < 0.0001; REST1RL: *D* = 0.237, *p* < 0.0001; REST2LR: *D* = 0.238, *p* < 0.0001; REST2RL: *D* = 0.250, *p* < 0.0001), indicating a systematic reduction of motion-related correlations following ICA-FIX denoising.

Machine-learning-based prediction analyses (fluid intelligence, age, and motion classification) were performed using linear models (ridge regression or ridge classification) as the primary approach. Generalisation performance was estimated using repeated nested CV. Specifically, 10 repetitions of fivefold outer CV were used, with an inner fivefold CV loop for hyperparameter tuning. Confound regression (framewise displacement and/or age) was performed in a CV-consistent manner, with confound models fit on training data only and applied to held-out test data. Prediction performance was summarised using out-of-sample coefficient of determination (R²) for regression tasks and classification accuracy and AUC for classification tasks. No statistical significance testing was applied to cross-validated prediction scores; instead, emphasis was placed on effect sizes and consistency across datasets and alignment conditions.

All analyses were repeated across multiple datasets to assess robustness and reproducibility. Key findings were reproduced in independent cohorts differing in scanner hardware, acquisition protocols, preprocessing pipelines, and age distributions. In addition, robustness analyses varying parcellation resolution, kernel functions, dimensionality reduction techniques, and preprocessing choices are reported in the Supplementary Material. All code used for data processing and analysis is available and fully deterministic given fixed random seeds, ensuring computational reproducibility.

### Reporting summary

Further information on research design is available in the Nature Portfolio Reporting Summary linked to this article.

## Supplementary information

Supplementary Information

Description of Additional Supplementary Files

Supplementary Data 1

Supplementary Data 2

Supplementary Data 3

Supplementary Data 4

Supplementary Data 5

Reporting Summary

## Data Availability

The data analysed in this study are publicly available from the Human Connectome Project (HCP) Open Access dataset, hosted on the AWS Open Data Registry by the WU-Minn HCP Consortium. Access to the data requires acceptance of the WU-Minn HCP Open Access Data Use Terms and registration via the ConnectomeDB portal (https://db.humanconnectome.org). The dataset can be programmatically retrieved using DataLad (version 0.12.2 or later), which provides fine-grained, version-controlled access to the HCP Open Access data without local hosting from the repository at https://github.com/datalad-datasets/human-connectome-project-openaccess. The HCP Open Access dataset is further available at: https://registry.opendata.aws/hcp-openaccess/. Further information on how to obtain the HCP-YA dataset can also be found at https://www.humanconnectome.org. The AOMIC PIOP1 (ds002785; 10.18112/openneuro.ds002785.v2.0.0) and PIOP2 (ds002790; 10.18112/openneuro.ds002790.v2.0.0) are available on OpenNeuro (see https://nilab-uva.github.io/AOMIC.github.io/), and may be accessed without restrictions (MIT License; https://github.com/NILAB-UvA/AOMIC.github.io/blob/master/LICENSE). Data from Cambridge Centre for Ageing Neuroscience dataset were retrieved from their official source at the CamCAN Data Portal (https://camcan-archive.mrc-cbu.cam.ac.uk/dataaccess/; following registration and acceptance of the data usage terms)^65,66^ and provided in the form of a DataLad dataset, a research data management solution providing data versioning, data transport, and provenance capture^67^. Source data for the plots in the figures are available in supplementary data files. Moreover, we provide a supplementary information file containing results for additional robustness checks and analyses. Any remaining information can be obtained from the corresponding author upon reasonable request.

## Code availability

All code written for this project is made publicly available here: https://github.com/juaml/procrustes_alignment_for_fc_gradients/. The code for this project was written and executed using Python (version 3.11.4) and its scientific ecosystem, in particular using the following software packages: numpy (version 1.25.2^72^), pandas (version 1.5.3^75^), scikit-learn (version 1.2.2^71^), scipy (version 1.11.2^76^), julearn (version 0.3.0^73^), junifer (version 0.0.3^77^), matplotlib (version 3.7.2^78^), and seaborn (version 0.12.2^79^). In addition, we forked the latest release version (0.1.10) of BrainSpace^16^ in order to adjust the code to make transformation matrices generated in Procrustes alignment available to the user. This fork is available at https://github.com/LeSasse/BrainSpace_release.
