# Supplementary material for: Individual identifiability following Procrustes alignment of functional gradients: effect of subspace dimensionality
Source: Commun Biol. 2026 Jan 10;9:231. doi: 10.1038/s42003-025-09509-3 (PMC12901060; doi:10.1038/s42003-025-09509-3)
Supplement: Supplementary file 1 — Supplementary Information [file 42003_2025_9509_MOESM1_ESM.pdf]

Individual Identifiability Following Procrustes Alignment of Functional Gradients: Effect of Subspace Dimensionality

Supplementary

July 15, 2025

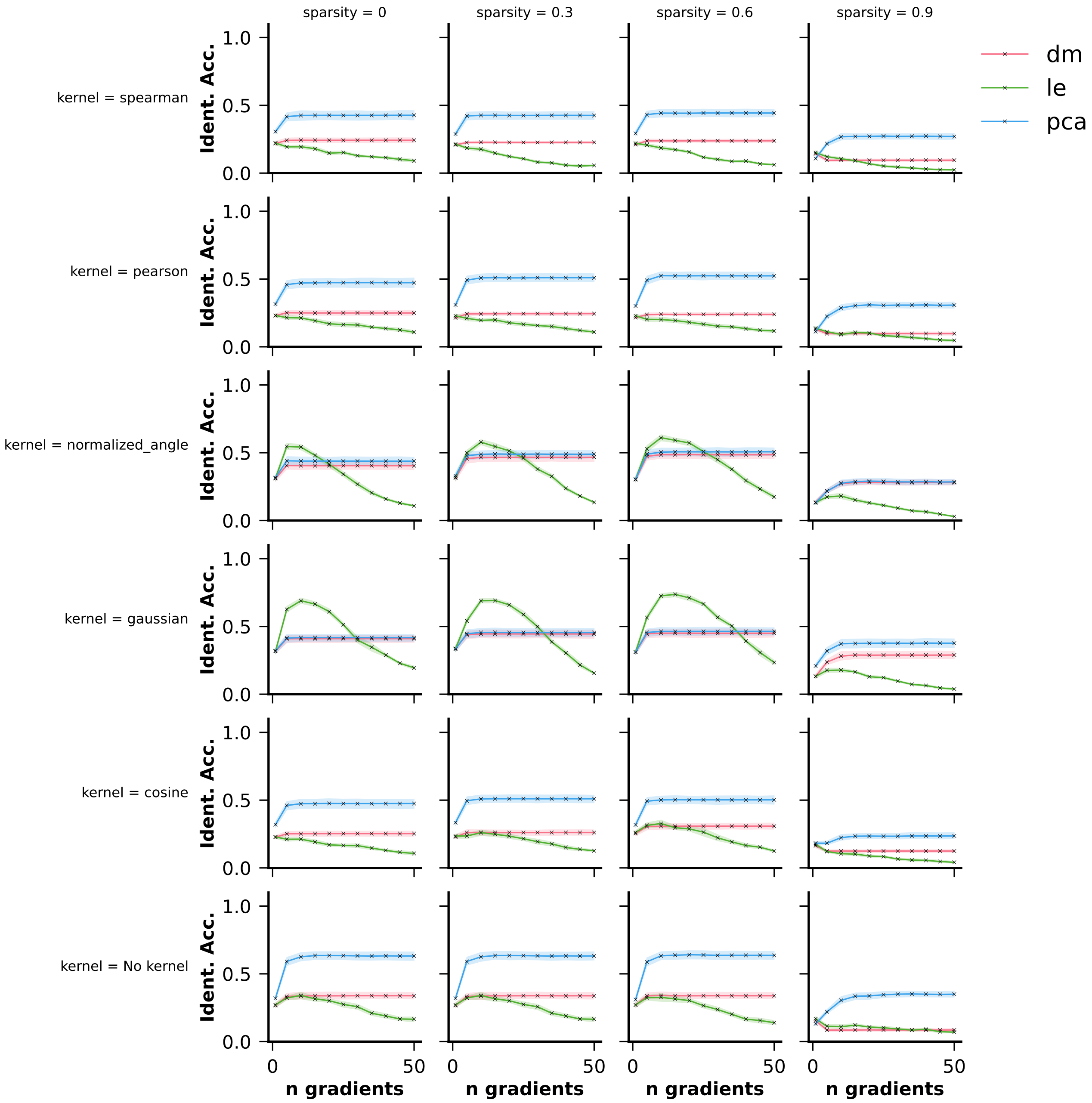

Figure S1. Identification accuracy (y-axis) across different kernels (rows), sparsities (columns), and dimensionality reduction approaches (hue) for varying numbers of gradients (x-axis) used in Procrustes alignment. FC gradients were extracted using the Schaefer 100 parcellation.

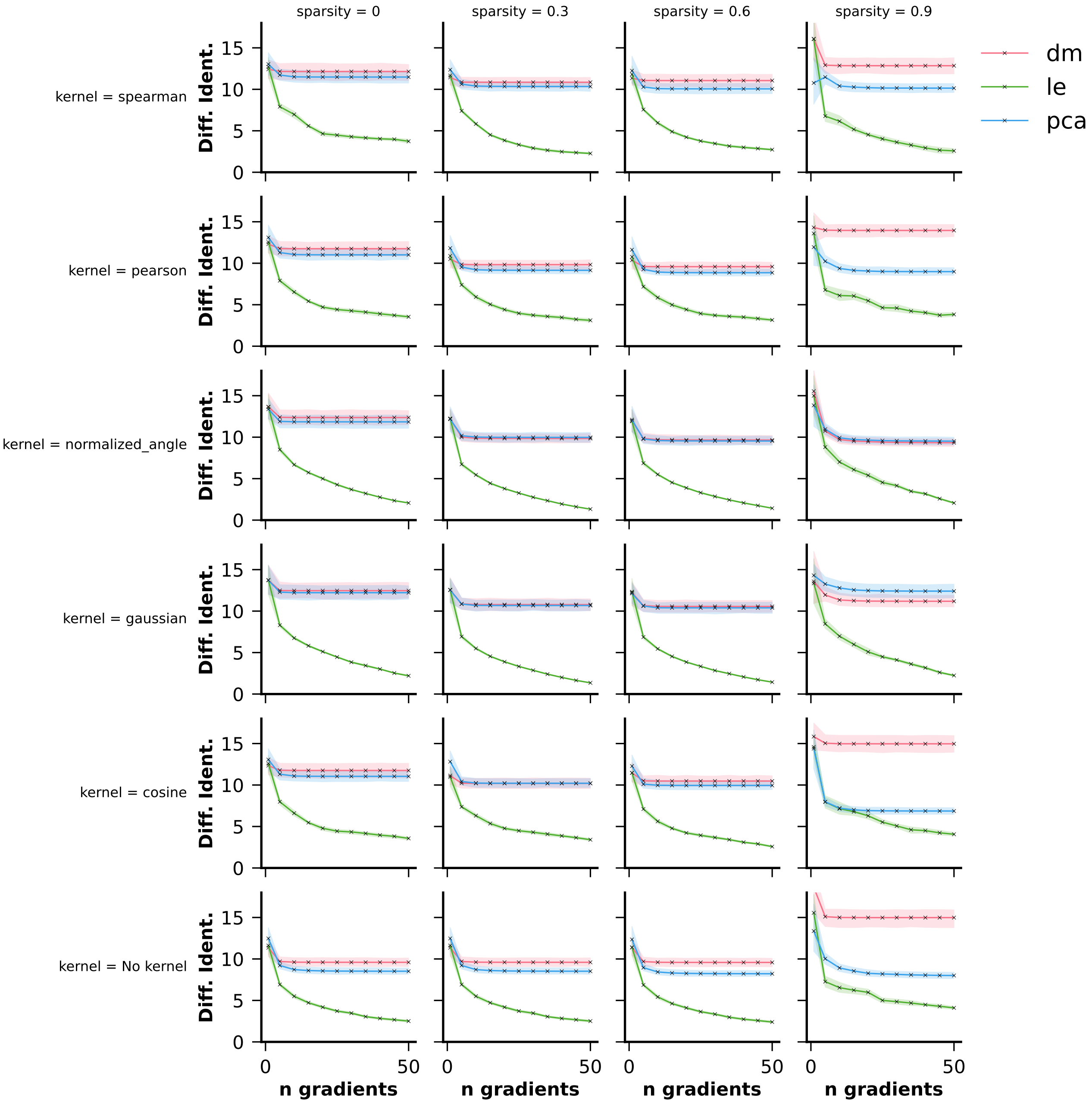

Figure S2. Differential identifiability (y-axis) across different kernels (rows), sparsities (columns), and dimensionality reduction approaches (hue) for varying numbers of gradients (x-axis) used in Procrustes alignment. FC gradients were extracted using the Schaefer 100 parcellation.

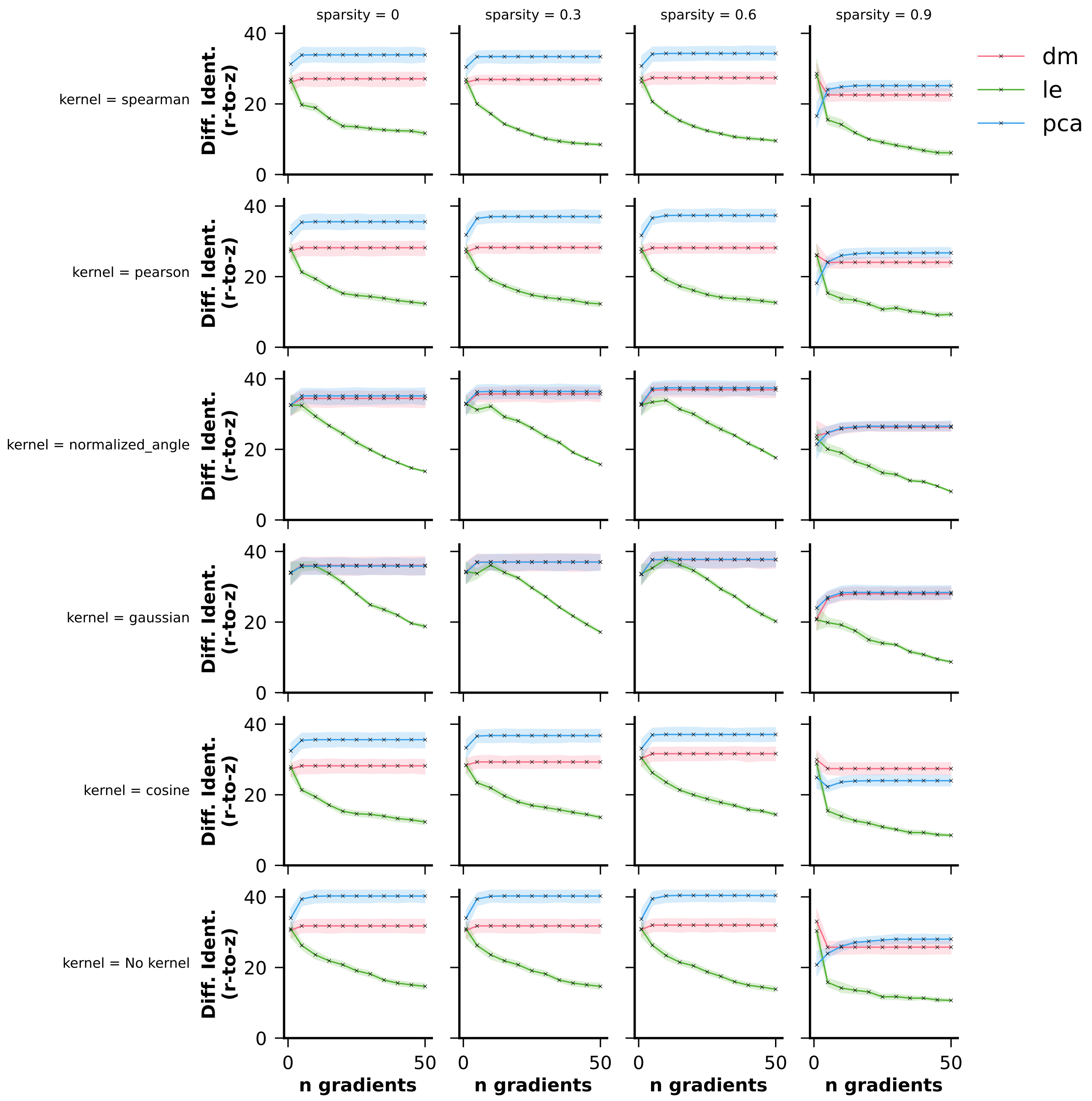

Figure S3. Differential identifiability (y-axis) after correlation values underwent Fisher's r-to-z transformation across different kernels (rows), sparsities (columns), and dimensionality reduction approaches (hue) for varying numbers of gradients (x-axis) used in Procrustes alignment. FC gradients were extracted using the Schaefer 100 parcellation.

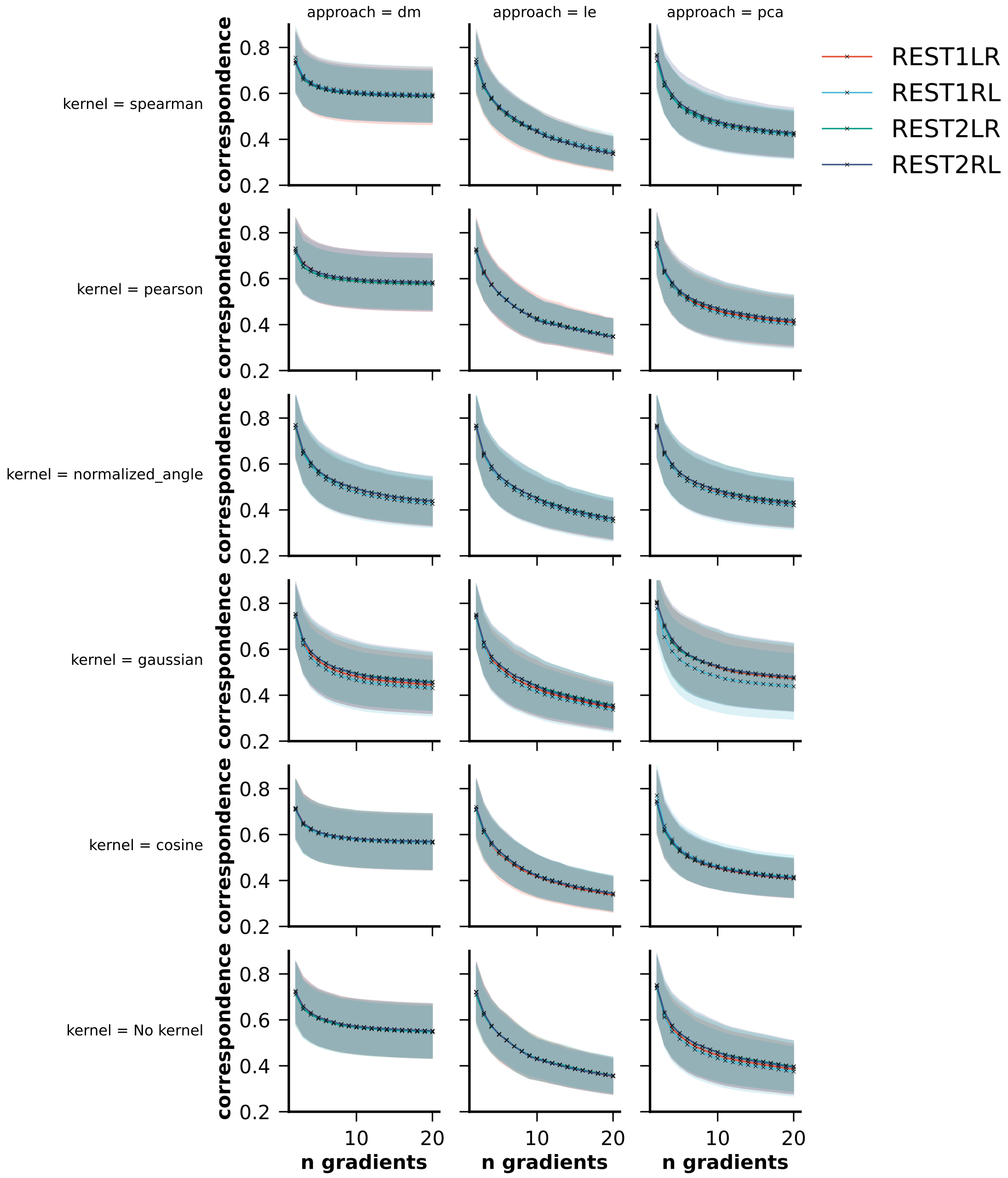

Figure S4. The correspondence between the unaligned and the aligned principal gradient per subject per session calculated using the transformation matrices. FC gradients were extracted using the Schaefer 100 parcellation and different kernels (rows) as well as dimensionality reduction approaches (columns).

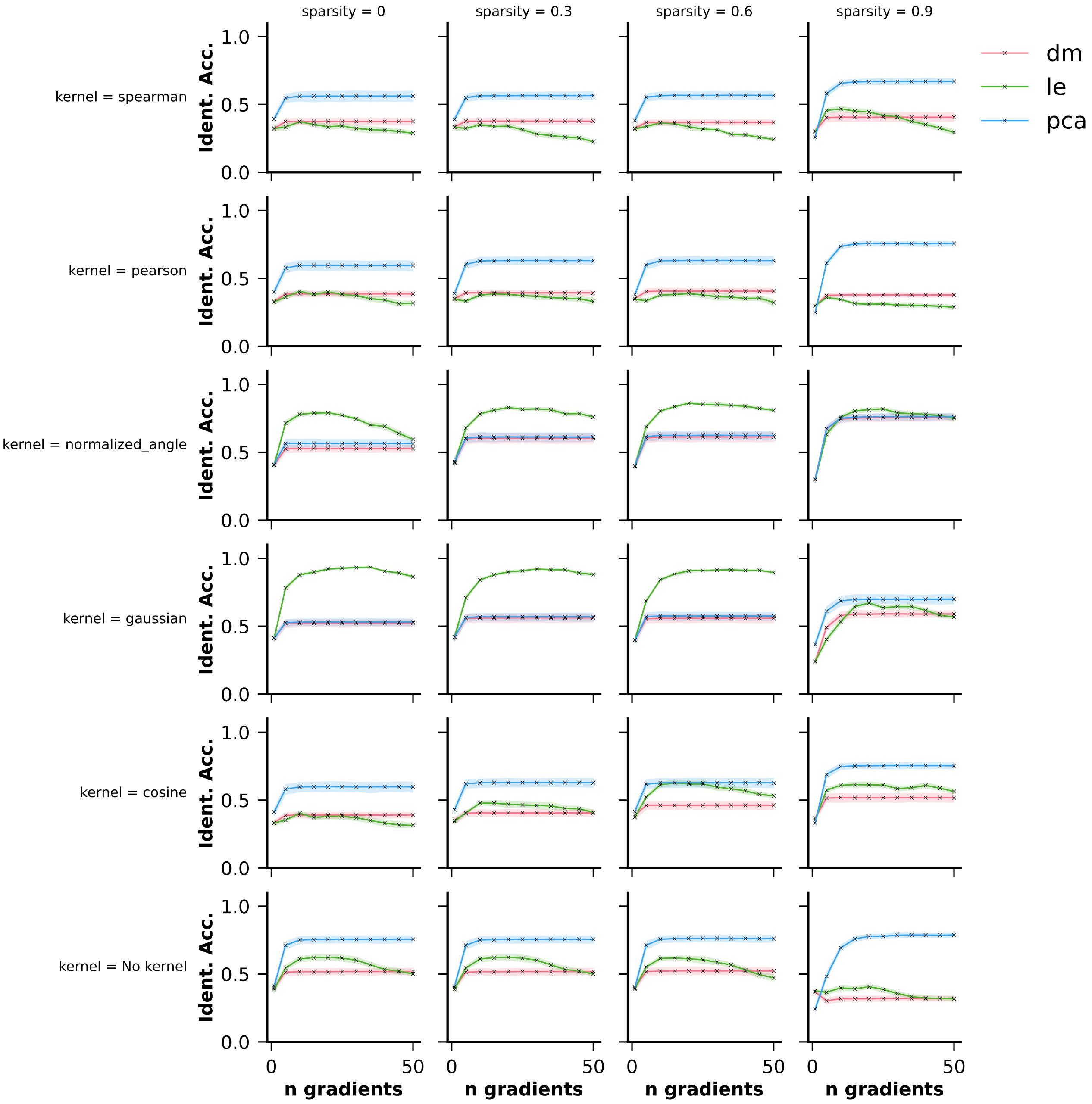

Figure S5. Identification accuracy (y-axis) across different kernels (rows), sparsities (columns), and dimensionality reduction approaches (hue) for varying numbers of gradients (x-axis) used in Procrustes alignment. FC gradients were extracted using the Schaefer 200 parcellation.

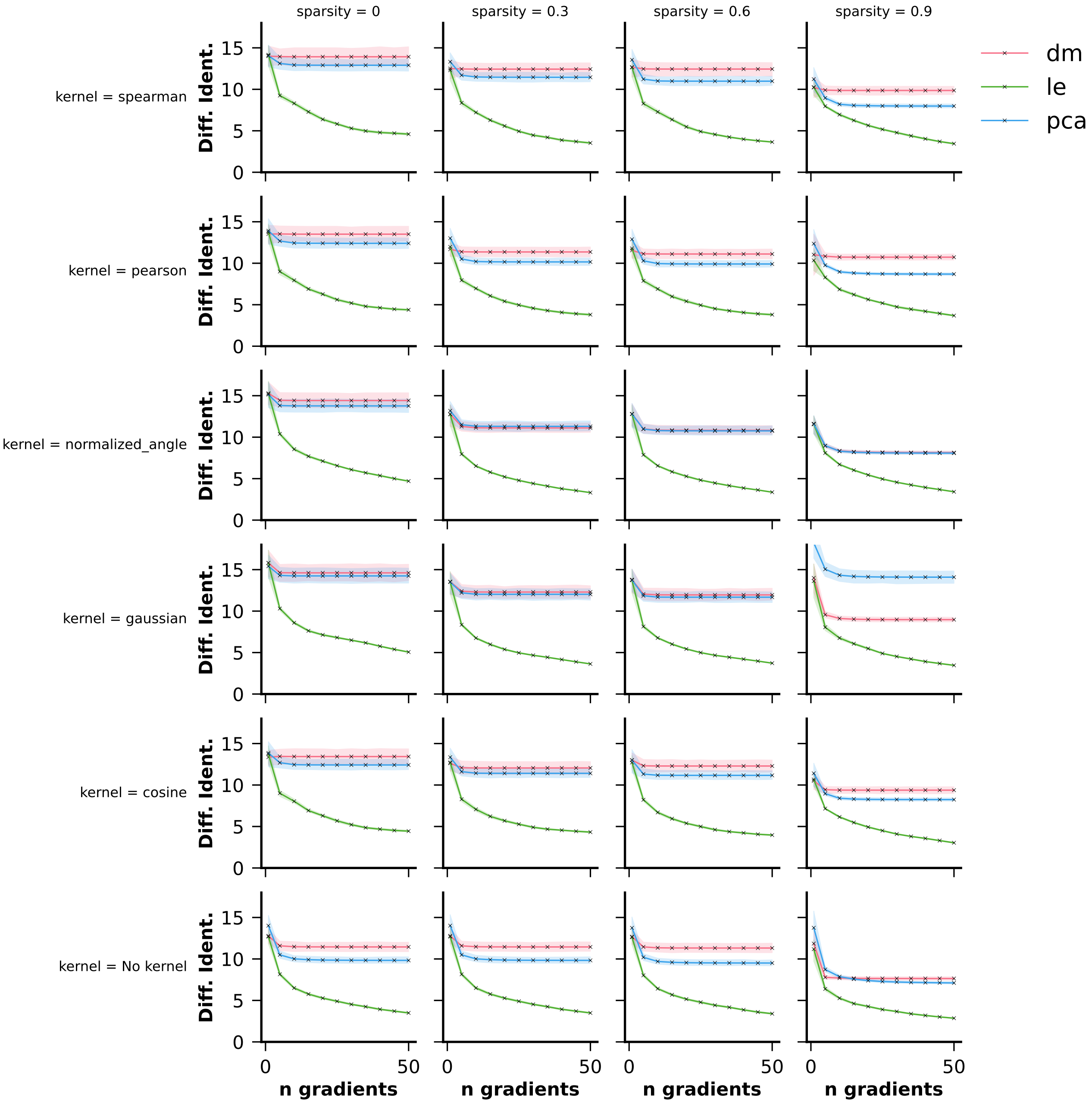

Figure S6. Differential identifiability (y-axis) across different kernels (rows), sparsities (columns), and dimensionality reduction approaches (hue) for varying numbers of gradients (x-axis) used in Procrustes alignment. FC gradients were extracted using the Schaefer 200 parcellation.

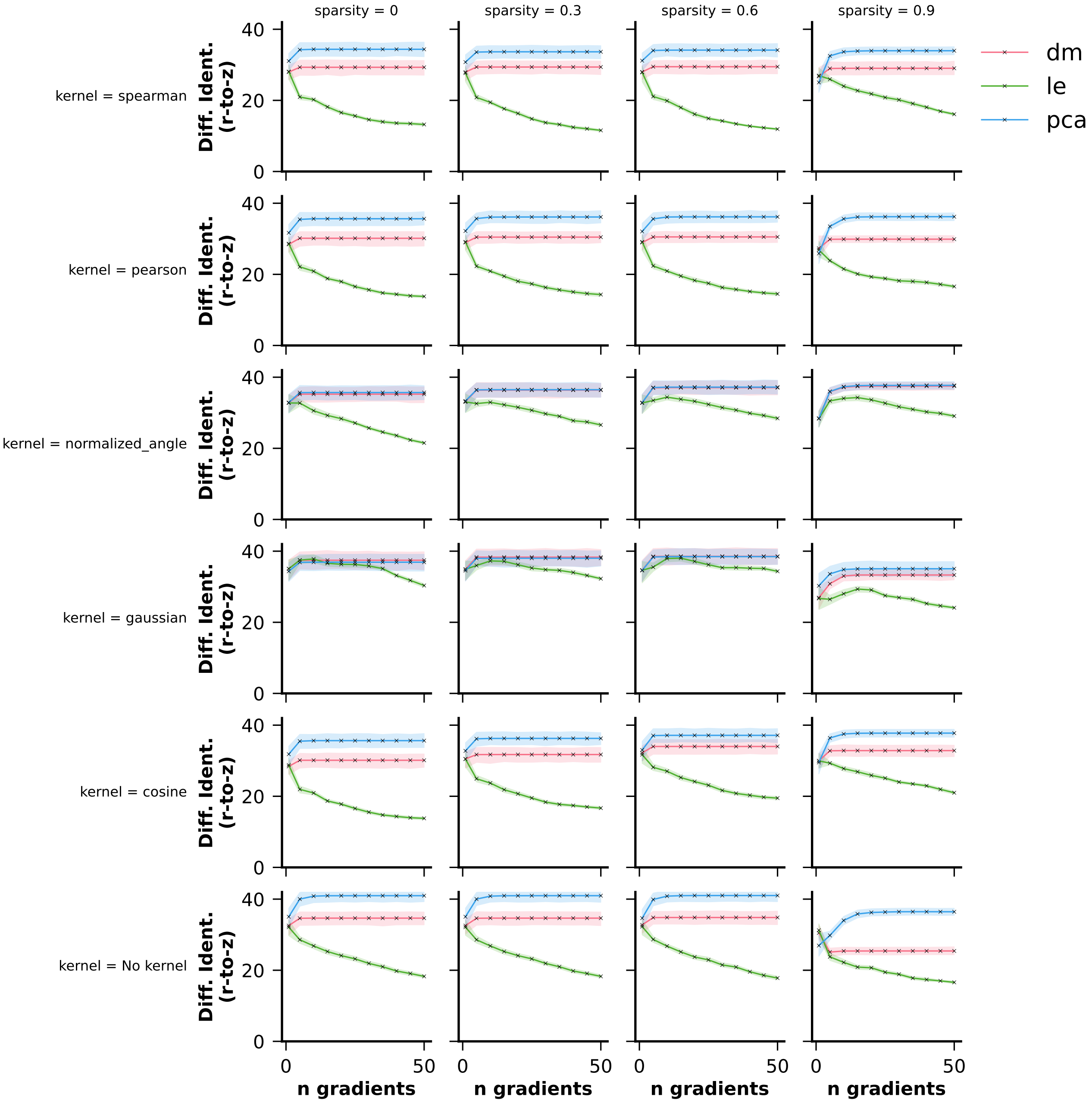

Figure S7. Differential identifiability (y-axis) after correlation values underwent Fisher's r-to-z transformation across different kernels (rows), sparsities (columns), and dimensionality reduction approaches (hue) for varying numbers of gradients (x-axis) used in Procrustes alignment. FC gradients were extracted using the Schaefer 200 parcellation.

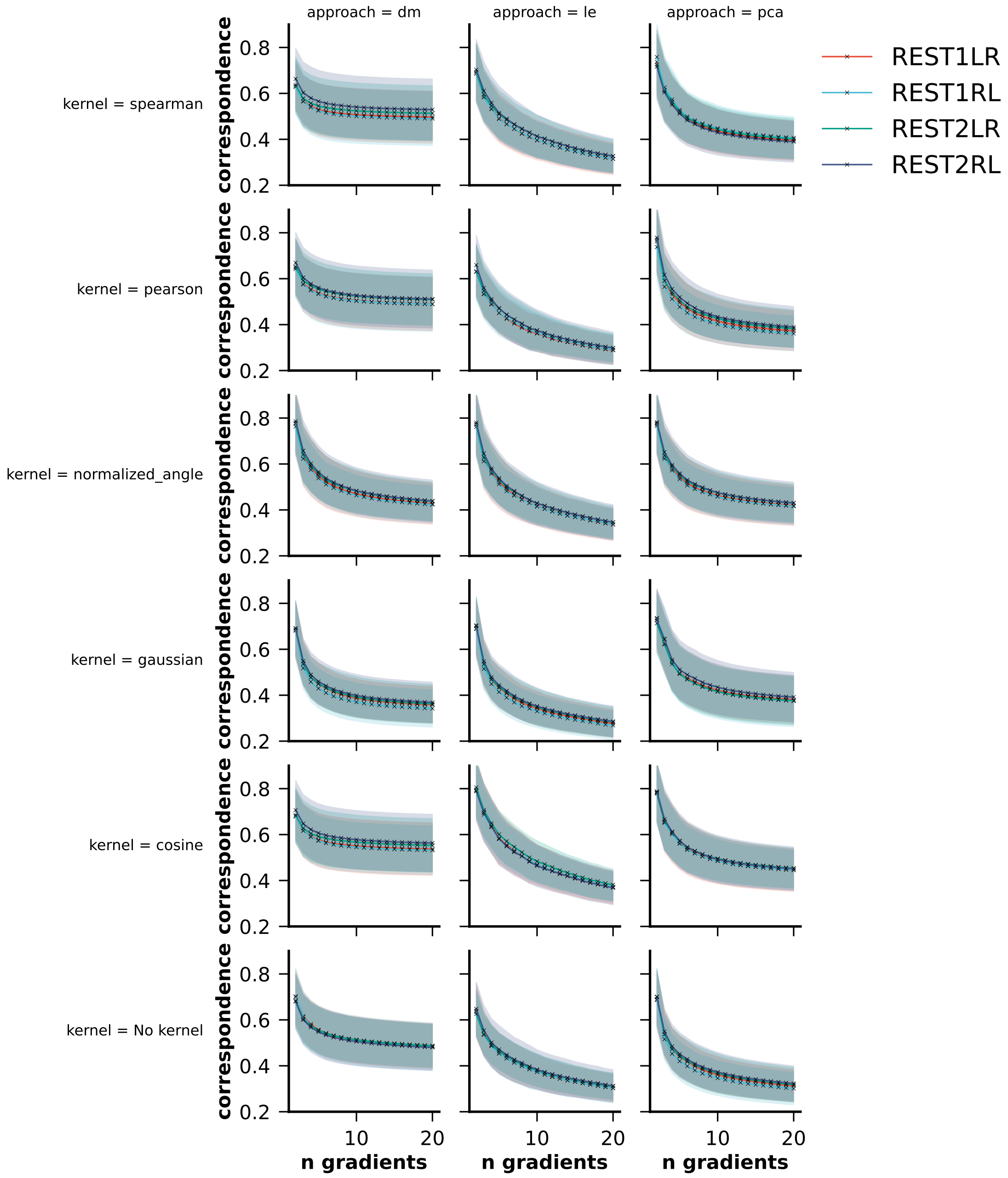

Figure S8. The correspondence between the unaligned and the aligned principal gradient per subject per session calculated using the transformation matrices. FC gradients were extracted using the Schaefer 200 parcellation and different kernels (rows) as well as dimensionality reduction approaches (columns).

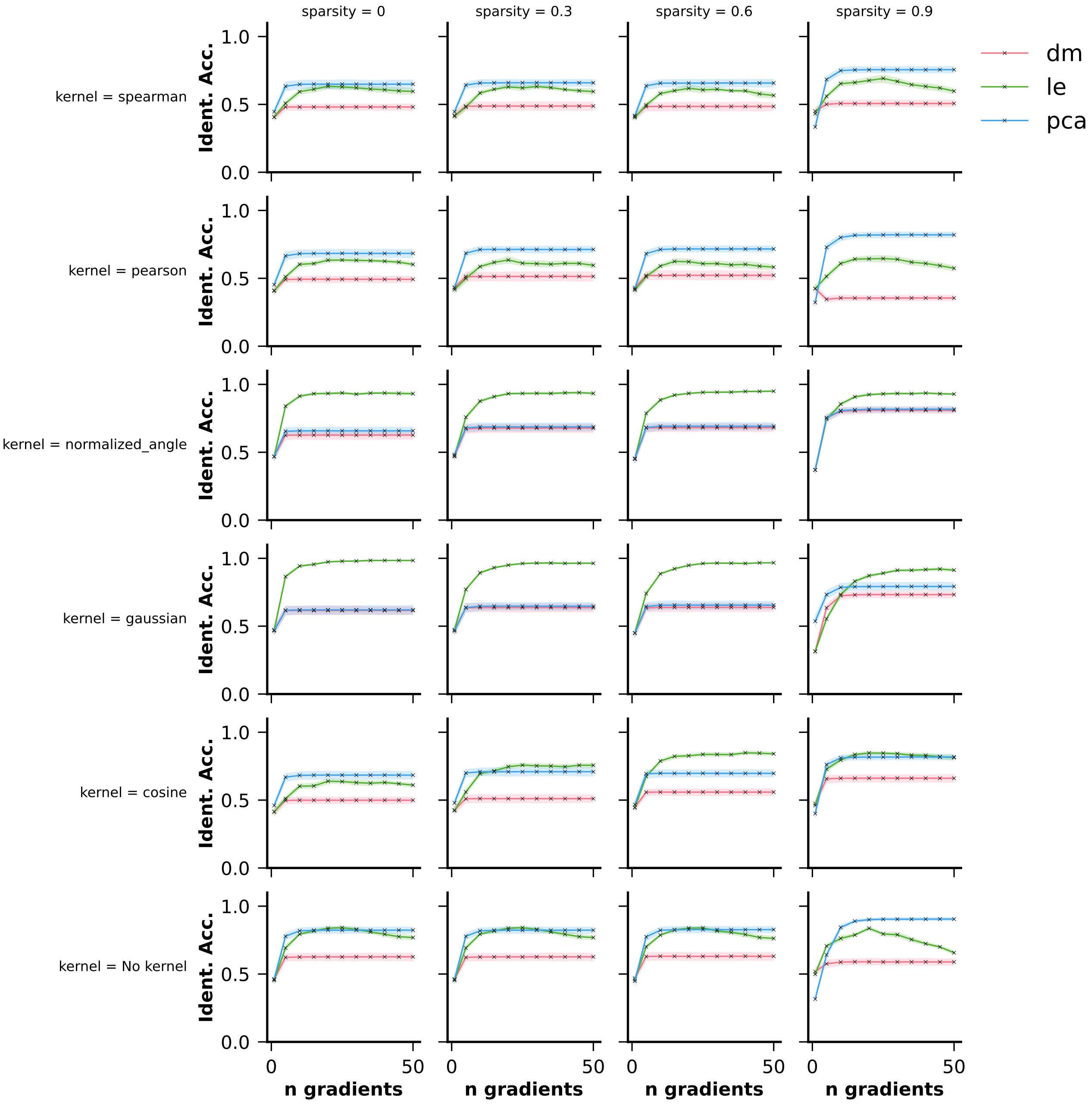

Figure S9. Identification accuracy (y-axis) across different kernels (rows), sparsities (columns), and dimensionality reduction approaches (hue) for varying numbers of gradients (x-axis) used in Procrustes alignment. FC gradients were extracted using the Schaefer 400 parcellation.

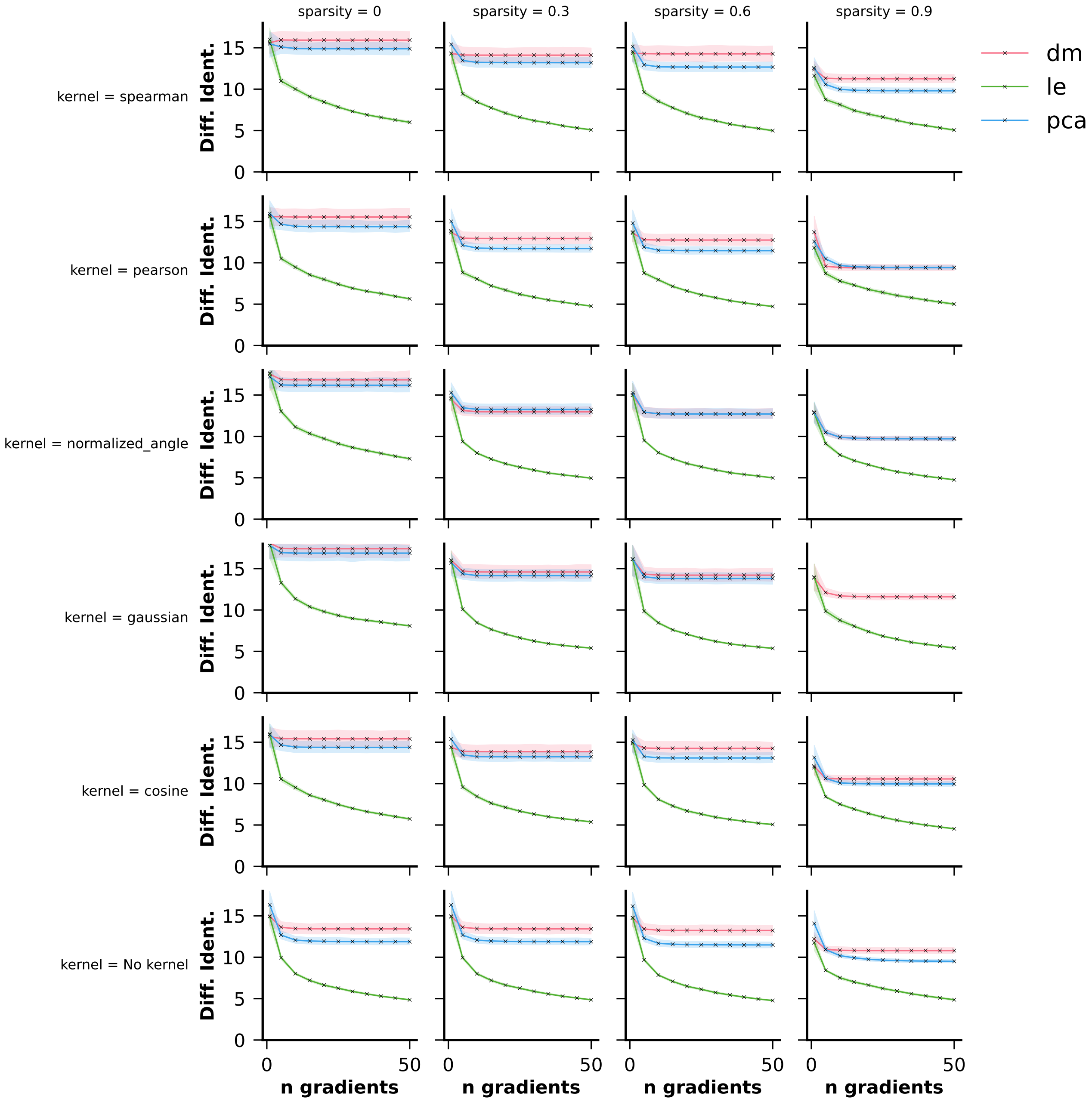

Figure S10. Differential identifiability (y-axis) across different kernels (rows), sparsities (columns), and dimensionality reduction approaches (hue) for varying numbers of gradients (x-axis) used in Procrustes alignment. FC gradients were extracted using the Schaefer 400 parcellation.

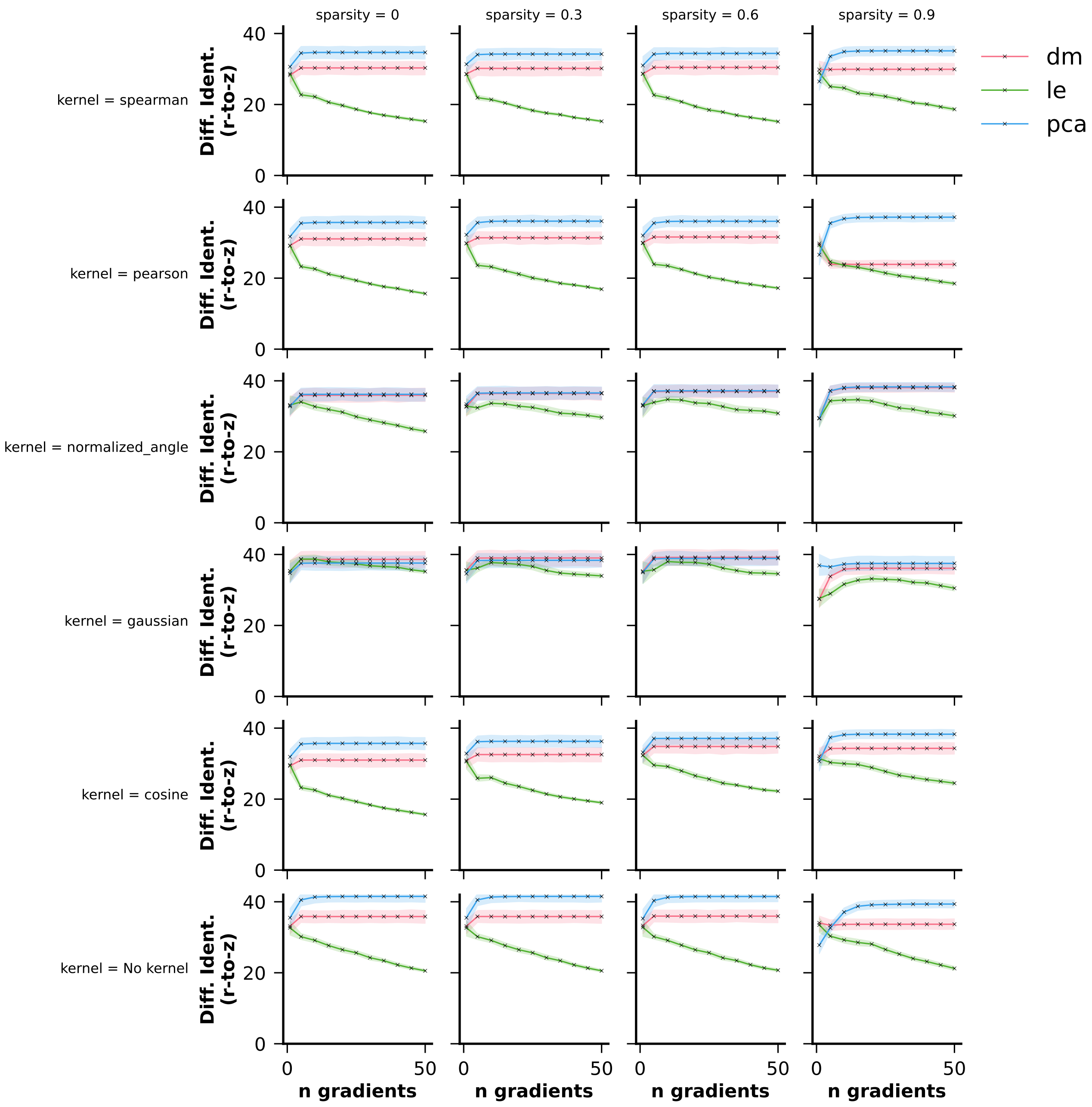

Figure S11. Differential identifiability (y-axis) after correlation values underwent Fisher's r-to-z transformation across different kernels (rows), sparsities (columns), and dimensionality reduction approaches (hue) for varying numbers of gradients (x-axis) used in Procrustes alignment. FC gradients were extracted using the Schaefer 400 parcellation.

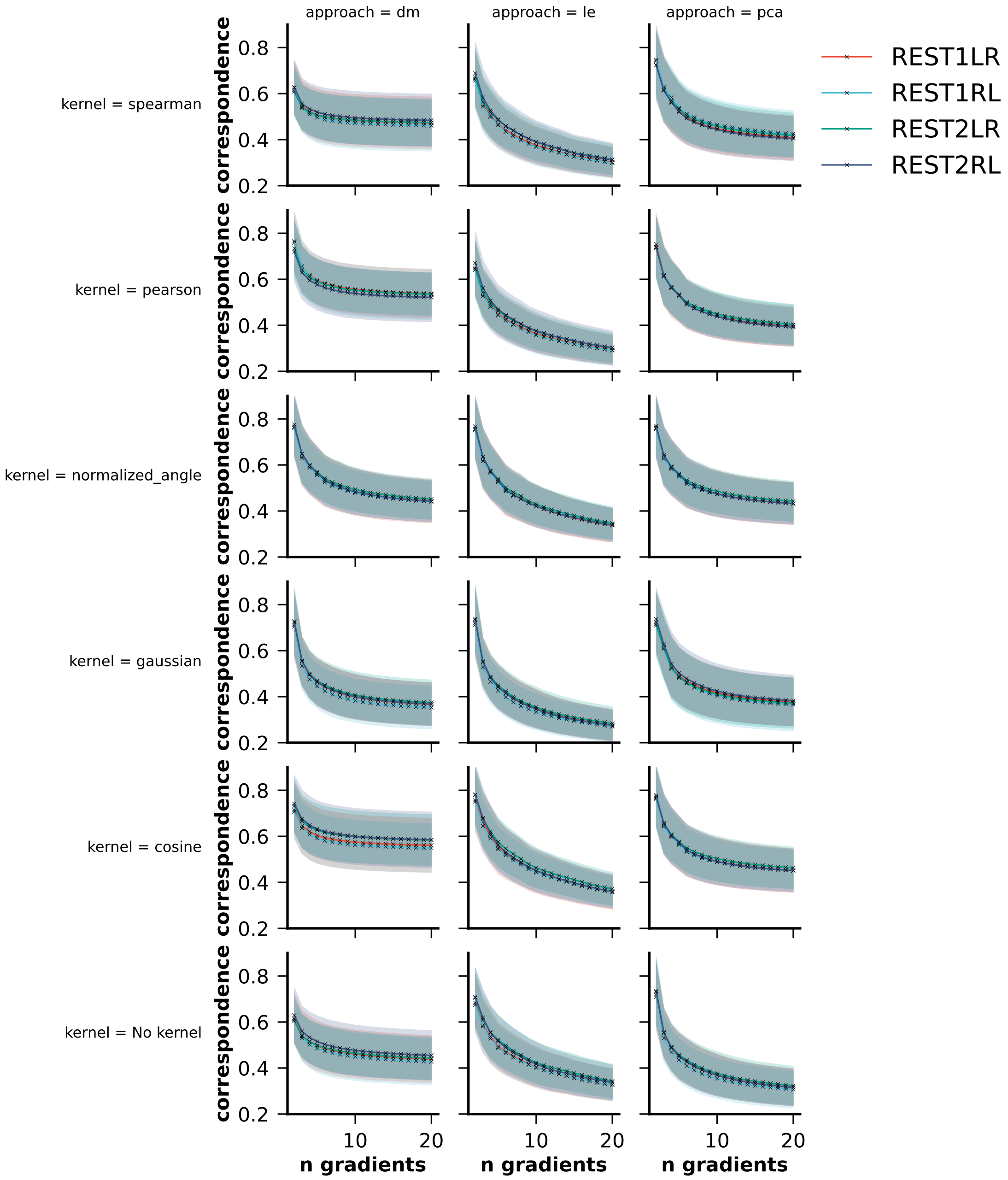

Figure S12. The correspondence between the unaligned and the aligned principal gradient per subject per session calculated using the transformation matrices. FC gradients were extracted using the Schaefer 400 parcellation and different kernels (rows) as well as dimensionality reduction approaches (columns).

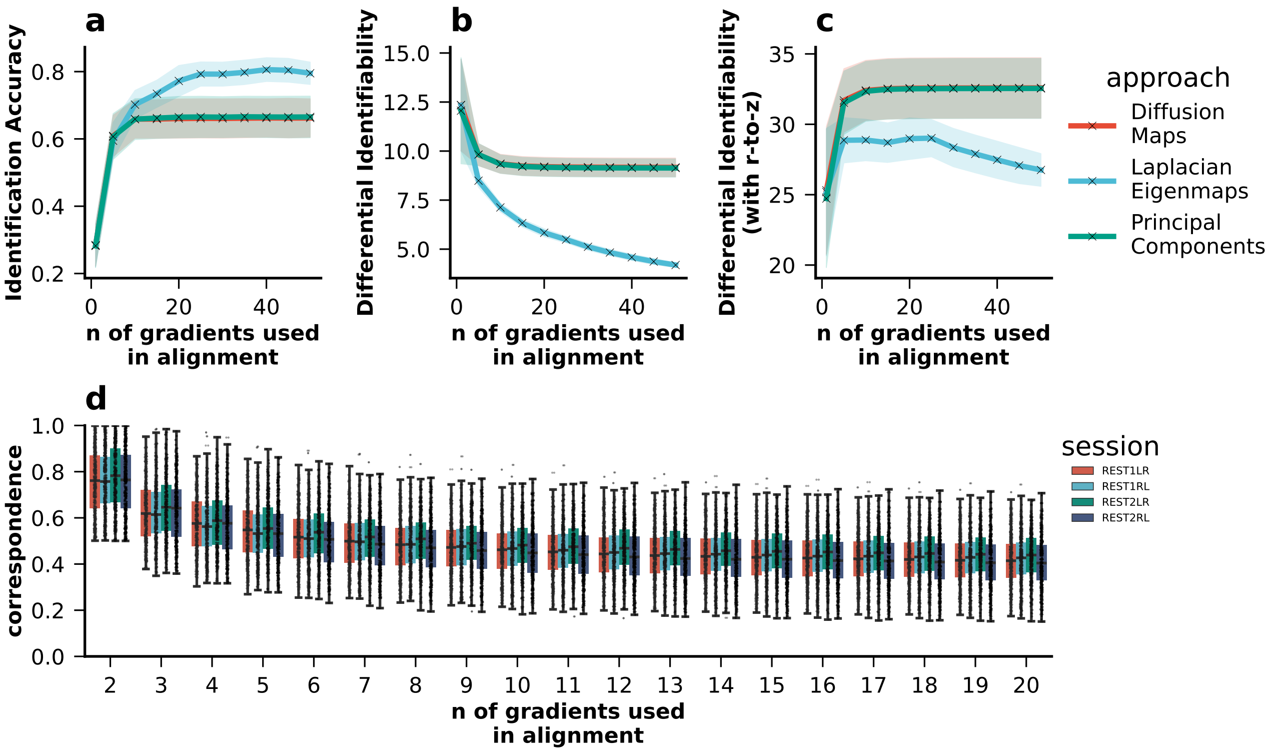

Figure S13. Impact of Procrustes alignment on **a)** identification accuracy and **b)** differential identifiability in the HCP-YA dataset without using ICA-FIX motion correction or motion regression. For each subject, gradients were extracted per session (kernel = normalized_angle; sparsity = 0.9). They were then aligned to the holdout reference gradient using Procrustes alignment. Identification accuracy and differential identifiability were calculated for each combination of sessions (NSessions= 4; NCombinations=6). **c)** Lastly, the correspondence between the unaligned and the aligned principal gradient per subject per session were calculated using the transformation matrices.

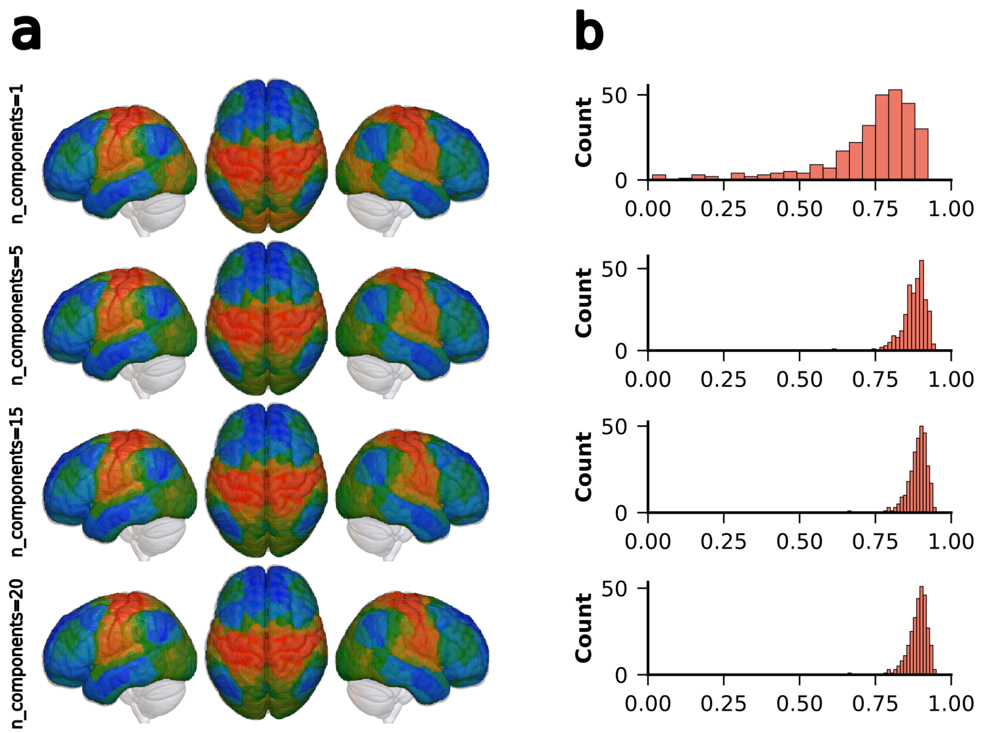

Figure S14. Group-level cortical patterns of the first principal gradient across alignment conditions. **a)** Spatial distribution of the average first gradient across subjects using the Schaefer 400 parcellation. The overall gradient structure remains stable across alignment conditions. This stability is expected at the group level, as averaging across subjects tends to smooth out individual variability and alignment-related differences. **b)** Correlation of aligned gradients with the reference gradient, showing increased similarity to the reference with a higher number of gradients used during alignment.

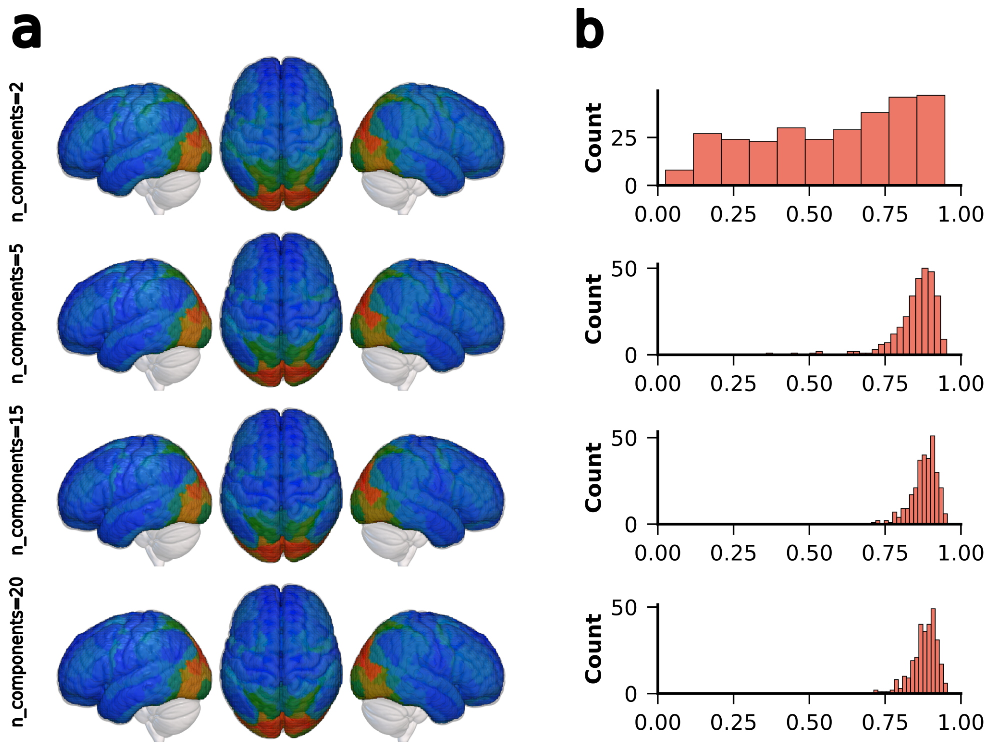

Figure S15. Group-level cortical patterns of the second principal gradient across alignment conditions. **a)** Spatial distribution of the average second gradient across subjects using the Schaefer 400 parcellation. The motor-to-visual axis remains largely consistent across alignment conditions, reflecting the relative stability of group-level gradients. **b)** Correlation of aligned second gradients with the reference gradient.

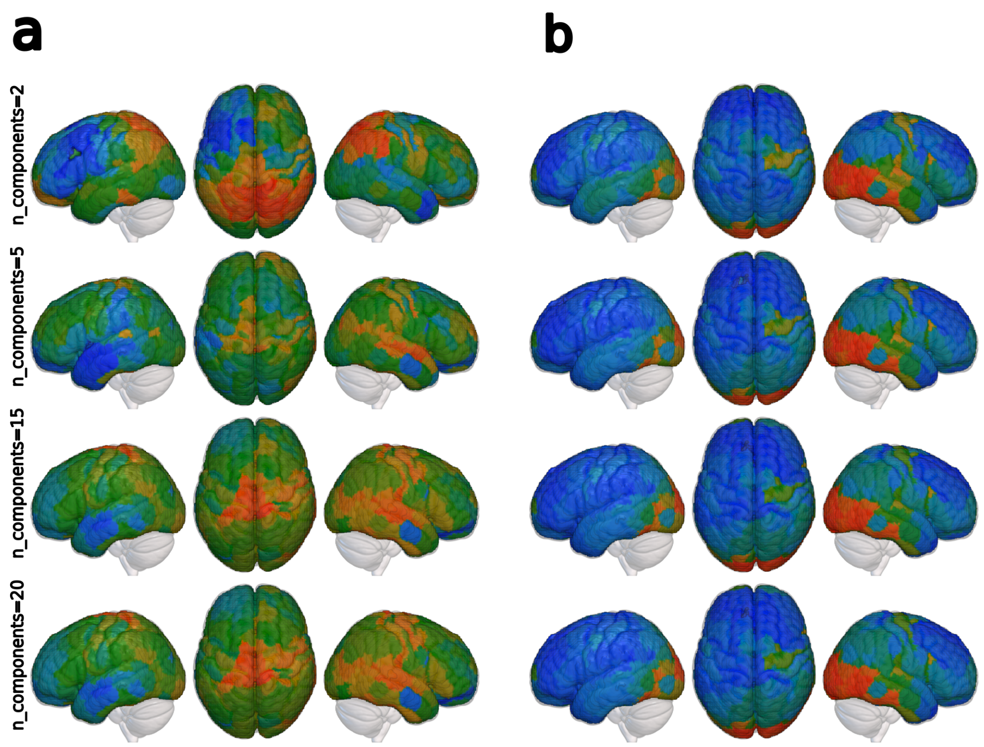

Figure S16. Individual-level cortical patterns of the first and second gradients under different alignment conditions in a low typicality subject. Cortical distributions of **a)** the first and **b)** the second gradient for a single subject with consistently low TFC scores across sessions in the HCP-YA dataset. The gradient patterns show notable alterations depending on the alignment condition, highlighting the influence of alignment at the individual level.

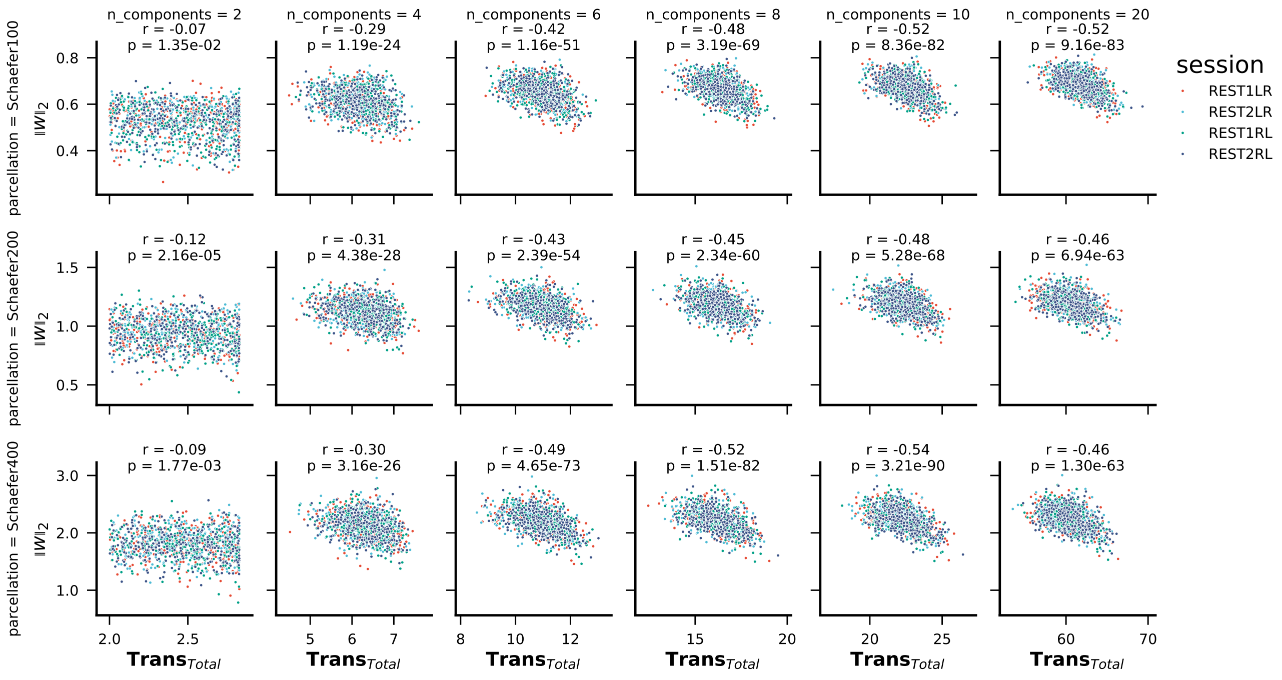

Figure S17. Relationship between $\|w\|_2$ and $Trans_{Total}$ for multiple parcellation granularities and numbers of gradients used in alignment (kernel = normalized_angle, approach = diffusion map embedding, sparsity = 0.9) in the HCP-YA dataset.

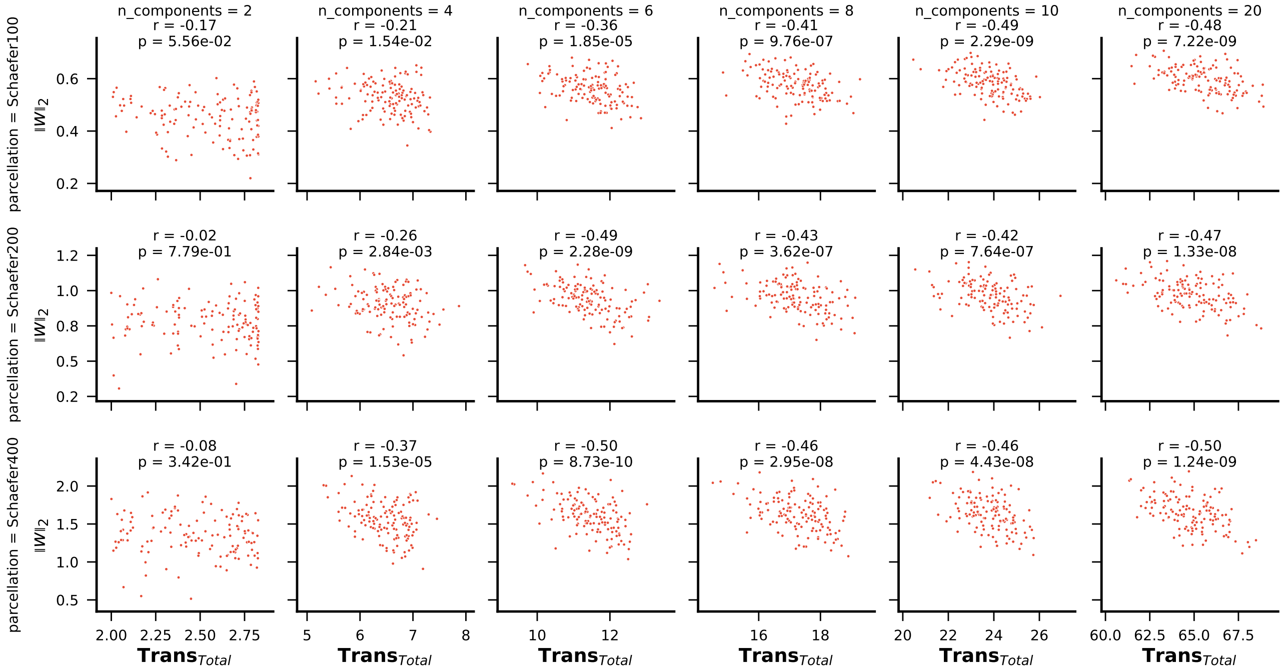

Figure S18. Relationship between $\|w\|_2$ and $Trans_{Total}$ for multiple parcellation granularities and numbers of gradients used in alignment (kernel = normalized_angle, approach = diffusion map embedding, sparsity = 0.9) in the AOMIC PIOP1 dataset.

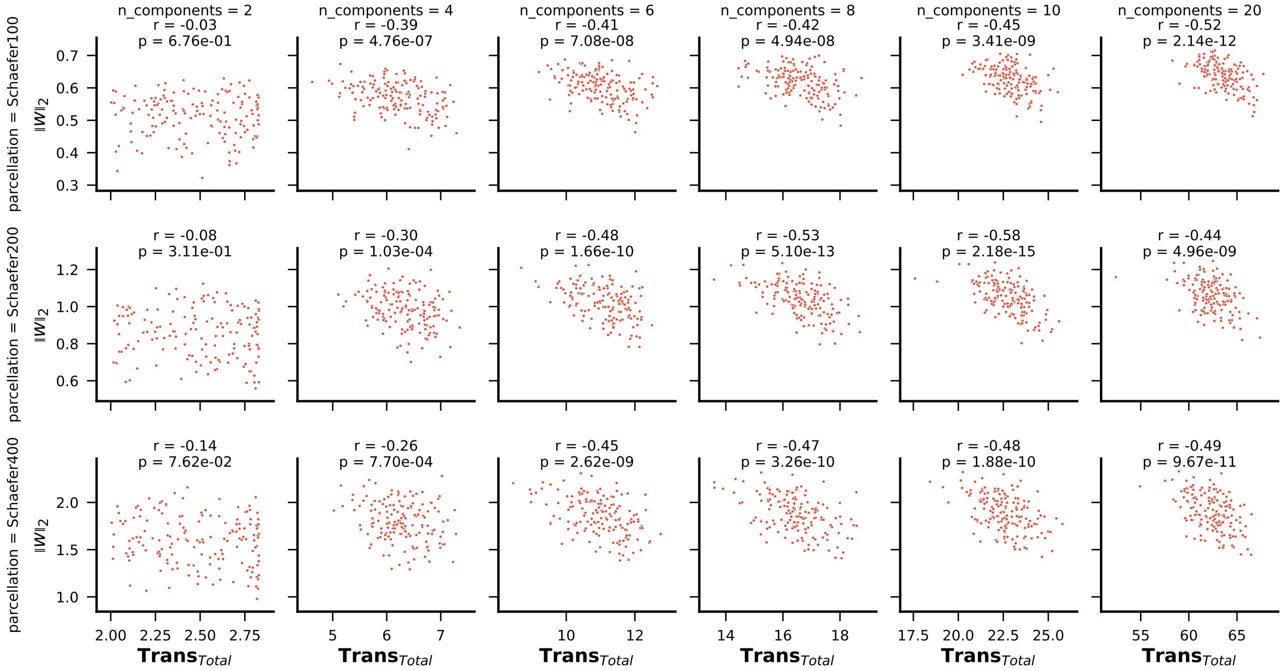

Figure S19. Relationship between $\|w\|_2$ and $Trans_{Total}$ for multiple parcellation granularities and numbers of gradients used in alignment (kernel = normalized_angle, approach = diffusion map embedding, sparsity = 0.9) in the AOMIC PIOP2 dataset.

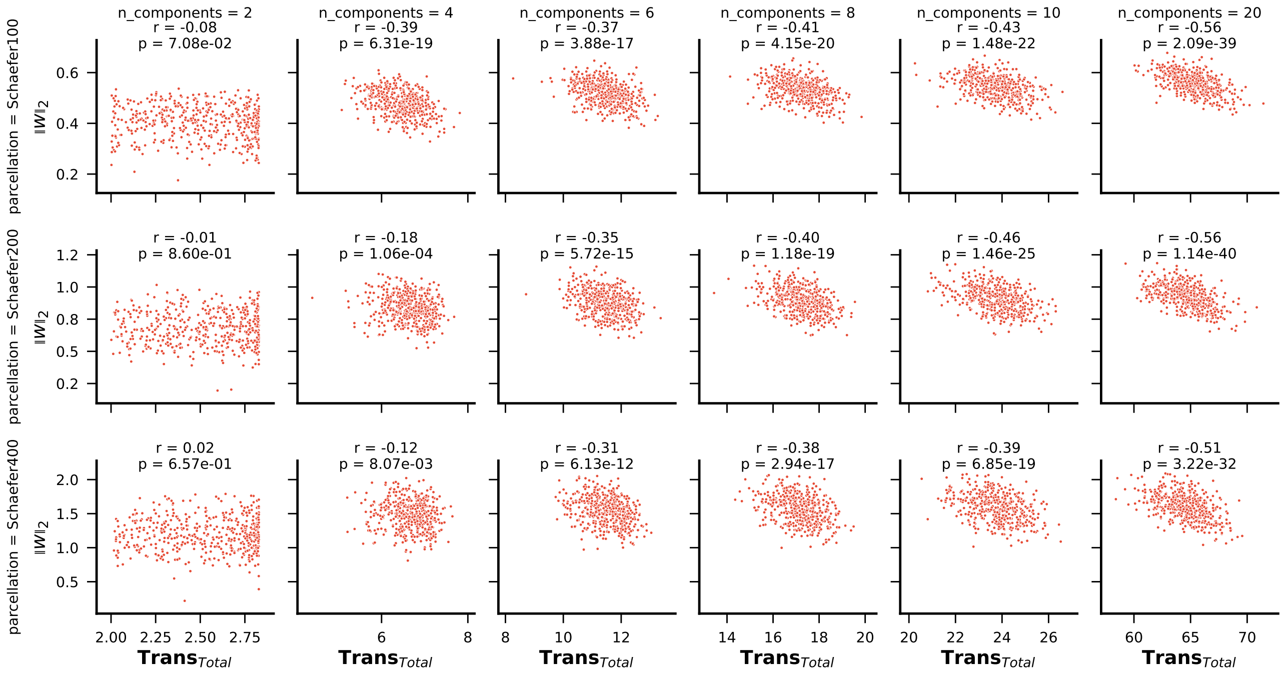

Figure S20. Relationship between $\|w\|_2$ and $Trans_{Total}$ for multiple parcellation granularities and numbers of gradients used in alignment (kernel = normalized_angle, approach = diffusion map embedding, sparsity = 0.9) in the Cam-CAN dataset.

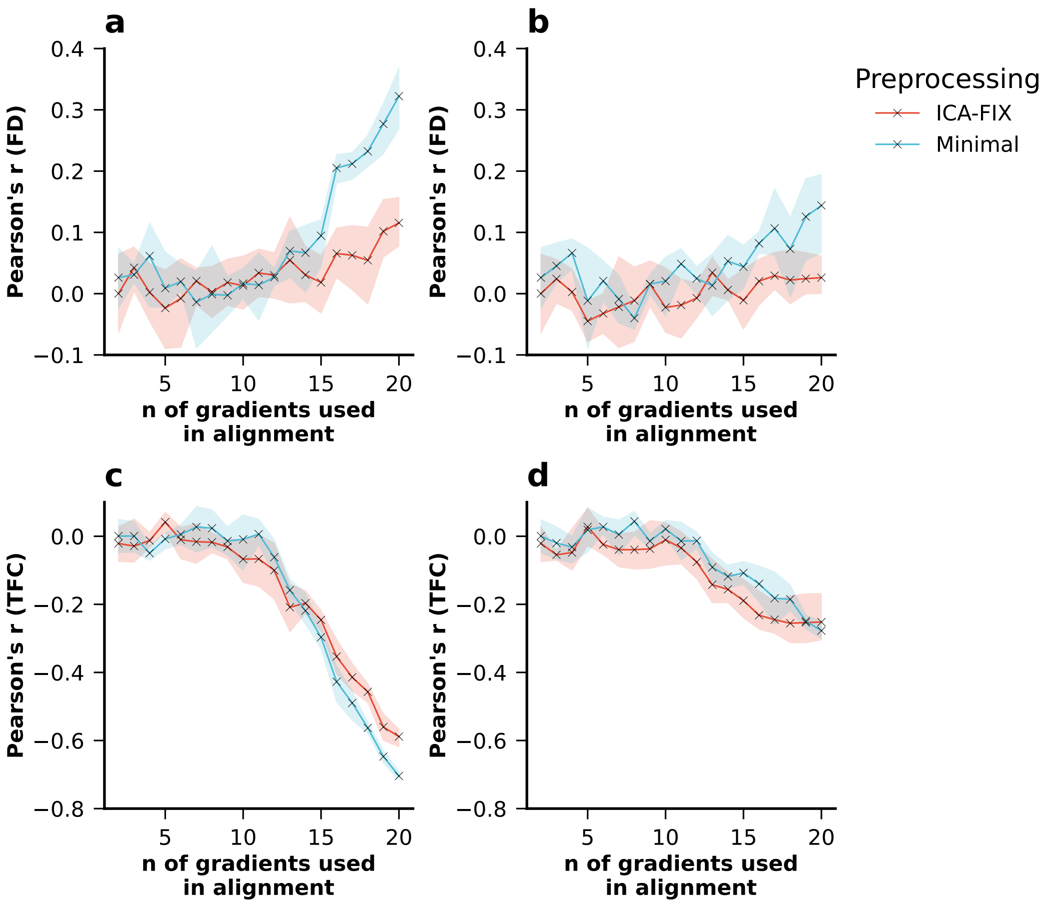

Figure S21. We aligned gradients in reverse order, starting with only the 20th gradient aligned to its holdout reference, then cumulatively adding gradients (20th and 19th, 20th to 18th, etc.) up to the full set of gradients 20 to 1. The plot shows the correlation between the aligned gradient set and motion metrics (mean framewise displacement) as a function of the number of gradients included in the alignment. Results reveal a gradual increase in motion correlation with the addition of more gradients, indicating that the accumulation of motion-related variance is not driven specifically by trailing gradients but arises cumulatively with each additional gradient included. This pattern supports the interpretation that the alignment process increasingly incorporates motion-related variance as the number of gradients increases, regardless of gradient rank. Notably, the increase in correlation is less pronounced compared to the original (leading-to-trailing) analysis, likely due to rank instability and reduced alignment reliability in the trailing gradients. a) Correlation between FD and the sum of absolute values of all elements in the transformation matrix and b) correlation between FD and the sum of absolute values of all elements in the first column (i.e. the column that determines the alignment of principal gradient) of the transformation matrix. c) Correlation between TFC and the sum of absolute values of all elements in the transformation matrix and d) correlation between TFC and the sum of absolute values of all elements in the first column (i.e. the column that determines the aligned principal gradient) of the transformation matrix.

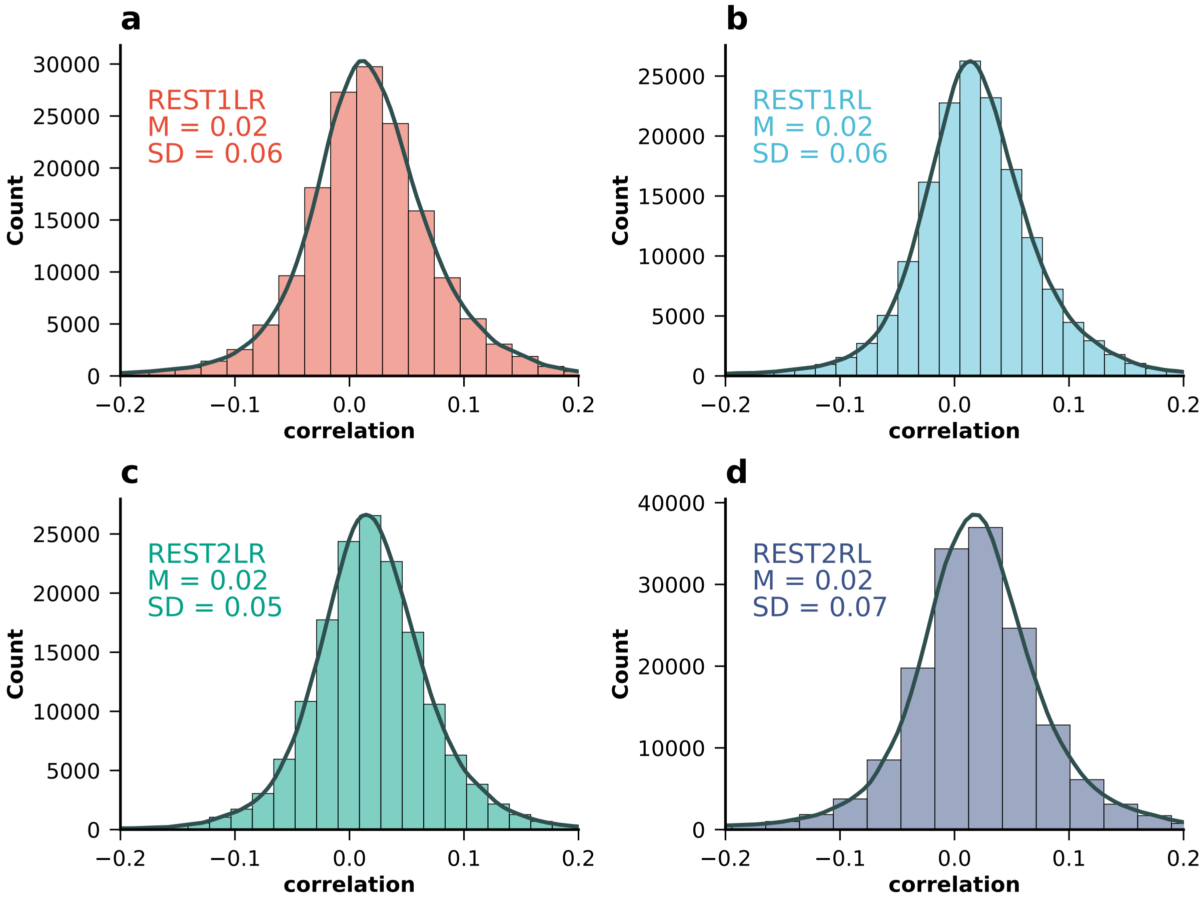

Figure S22. Distribution of correlations between each subjects' ROI time series and FD time series for all four resting state fMRI sessions in the HCP-YA dataset without using ICA-FIX motion correction or motion regression: **a)** REST1LR, **b)** REST1RL, **c)** REST2LR, and **d)** REST2RL.

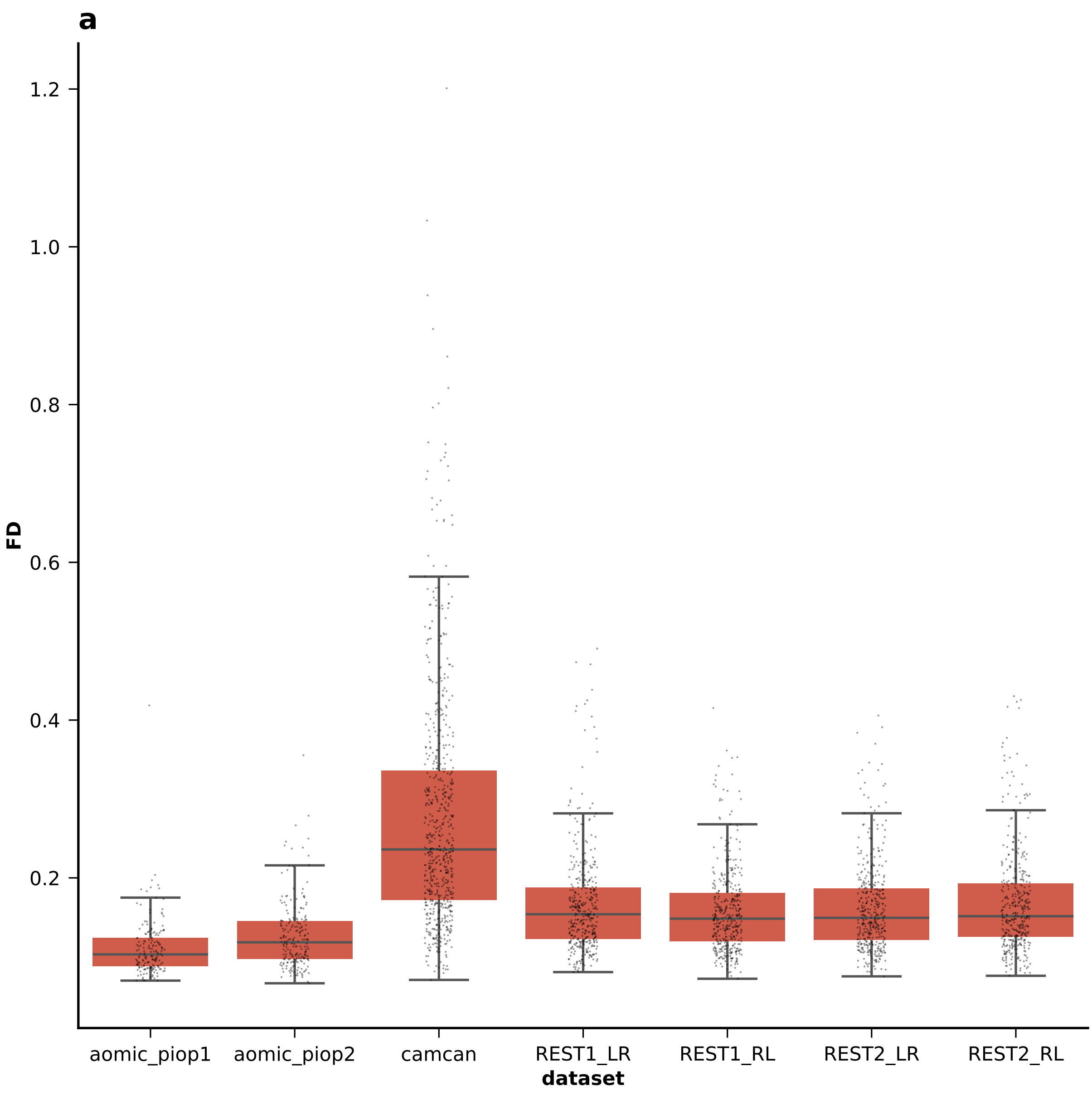

Figure S23. Distribution of FD values for all datasets
