# Supplementary material for: Individual identifiability following Procrustes alignment of functional gradients: effect of subspace dimensionality
Source: Commun Biol. 2026 Jan 10;9:231. doi: 10.1038/s42003-025-09509-3 (PMC12901060; doi:10.1038/s42003-025-09509-3)
Supplement: Supplementary file 2 — Description of Additional Supplementary Files [file 42003_2025_9509_MOESM2_ESM.docx]

**Description of Additional Supplementary File**

File name: Supplementary Data 1
Description: [Supplementary Data 1] Results of the identification accuracy and subject identifiability analysis as well as regression of intelligence and classification of motion (high vs low) across all datasets. Source Data For Figs 1a and b, 6, 7.

File name: Supplementary Data 2
Description: [Supplementary Data 2] Results obtained from inspecting the Procrustes (rotation matrix) i.e. TransTotal, L2 of singular values, and the L2 of subspace angles across all datasets. Source Data For Figs 1c, 2, 4a, 4d-i, 5a, 5c-h.

File name: Supplementary Data 3
Description: [Supplementary Data 3] Subjectwise framewise displacement values across all the datasets. Source Data For Figs 2a-d, 4d-f, 5e,c, and g.

File name: Supplementary Data 4
Description: [Supplementary Data 4] Typicality of functional connectivity of subjects across all datasets used. Source Data For Figs 2e-h, 4g-i, 5d,f,h.

File name: Supplementary Data 5
Description: [Supplementary Data 5] Framewise displacement timeseries per brain region per subject. Source Data For Figs 3, 4b and c, 5b.
