# Supplementary material for: Individual identifiability following Procrustes alignment of functional gradients: effect of subspace dimensionality
Source: Commun Biol. 2026 Jan 10;9:231. doi: 10.1038/s42003-025-09509-3 (PMC12901060; doi:10.1038/s42003-025-09509-3)
Supplement: Supplementary file 8 — Reporting Summary [file 42003_2025_9509_MOESM8_ESM.pdf]

nature portfolio

Corresponding author(s): Kaustubh Patil, Leonard Sasse

Last updated by author(s): 15.07.2025



# Reporting Summary

Nature Portfolio wishes to improve the reproducibility of the work that we publish. This form provides structure for consistency and transparency in reporting. For further information on Nature Portfolio policies, see our Editorial Policies and the Editorial Policy Checklist.

## Statistics

For all statistical analyses, confirm that the following items are present in the figure legend, table legend, main text, or Methods section.

| n/a | Confirmed | |
|---|---|---|
| ☐ | ☒ | The exact sample size (*n*) for each experimental group/condition, given as a discrete number and unit of measurement |
| ☐ | ☒ | A statement on whether measurements were taken from distinct samples or whether the same sample was measured repeatedly |
| ☐ | ☒ | The statistical test(s) used AND whether they are one- or two-sided<br>*Only common tests should be described solely by name; describe more complex techniques in the Methods section.* |
| ☒ | ☐ | A description of all covariates tested |
| ☒ | ☐ | A description of any assumptions or corrections, such as tests of normality and adjustment for multiple comparisons |
| ☐ | ☒ | A full description of the statistical parameters including central tendency (e.g. means) or other basic estimates (e.g. regression coefficient) AND variation (e.g. standard deviation) or associated estimates of uncertainty (e.g. confidence intervals) |
| ☐ | ☒ | For null hypothesis testing, the test statistic (e.g. *F*, *t*, *r*) with confidence intervals, effect sizes, degrees of freedom and *P* value noted<br>*Give P values as exact values whenever suitable.* |
| ☒ | ☐ | For Bayesian analysis, information on the choice of priors and Markov chain Monte Carlo settings |
| ☒ | ☐ | For hierarchical and complex designs, identification of the appropriate level for tests and full reporting of outcomes |
| ☐ | ☒ | Estimates of effect sizes (e.g. Cohen's *d*, Pearson's *r*), indicating how they were calculated |

*Our web collection on statistics for biologists contains articles on many of the points above.*

## Software and code

Policy information about availability of computer code

| | |
|---|---|
| Data collection | Further information on how to obtain the HCP-YA dataset can be obtained at https://www.humanconnectome.org/. The AOMIC PIOP1 and PIOP2 are available on OpenNeuro (see https://nilab-uva.github.io/AOMIC.github.io/). Data from Cambridge Centre for Ageing Neuroscience dataset were retrieved from their official source at the CamCAN Data Portal (https://camcan-archive.mrc-cbu.cam.ac.uk/dataaccess/) (Shafto et al., 2014; Taylor et al., 2017) and provided in the form of a DataLad dataset, a research data management solution providing data versioning, data transport, and provenance capture (Halchenko et al., 2021). |
| Data analysis | All code written for this project is made publicly available here: https://github.com/juaml/procrustes_alignment_for_fc_gradients/. The code for this project was written and executed using Python (version 3.11.4) and its scientific ecosystem, in particular using the following software packages: numpy (version 1.25.2; (Harris et al., 2020)), pandas (version 1.5.3; (McKinney, 2010)), scikit-learn (version 1.2.2; (Pedregosa et al., 2011)), scipy (version 1.11.2; (Virtanen et al., 2020)), julearn (version 0.3.0; (Hamdan et al., 2024)), junifer (version 0.0.3; (Mandal et al., 2023)), matplotlib (version 3.7.2; (Hunter, 2007)), and seaborn (version 0.12.2; (Waskom, 2021)). In addition, we forked the latest release version (0.1.10) of BrainSpace (Vos de Wael et al., 2020) in order to adjust the code to make transformation matrices generated in Procrustes alignment available to the user. This fork is available at https://github.com/LeSasse/BrainSpace_release. |

For manuscripts utilizing custom algorithms or software that are central to the research but not yet described in published literature, software must be made available to editors and reviewers. We strongly encourage code deposition in a community repository (e.g. GitHub). See the Nature Portfolio guidelines for submitting code & software for further information.

## Data

Policy information about availability of data

All manuscripts must include a data availability statement. This statement should provide the following information, where applicable:

- Accession codes, unique identifiers, or web links for publicly available datasets
- A description of any restrictions on data availability
- For clinical datasets or third party data, please ensure that the statement adheres to our policy

Further information on how to obtain the HCP-YA dataset can be obtained at https://www.humanconnectome.org/. The AOMIC PIOP1 and PIOP2 are available on OpenNeuro (see https://nilab-uva.github.io/AOMIC.github.io/). Data from Cambridge Centre for Ageing Neuroscience dataset were retrieved from their official source at the CamCAN Data Portal (https://camcan-archive.mrc-cbu.cam.ac.uk/dataaccess/) (Shafto et al., 2014; Taylor et al., 2017) and provided in the form of a DataLad dataset, a research data management solution providing data versioning, data transport, and provenance capture (Halchenko et al., 2021).

## Research involving human participants, their data, or biological material

Policy information about studies with human participants or human data. See also policy information about sex, gender (identity/presentation), and sexual orientation and race, ethnicity and racism.

| | |
|---|---|
| Reporting on sex and gender | HCP-YA: The average age of participants is 28.74 years with a standard deviation of 3.82 years, covering an age range from 22 to 37 years. AOMIC-PIOP1: The sample has a mean age of 22.09 years with an SD of 1.77 years, and participant ages range from 18 to 26 years. AOMIC-PIOP2: Participants have an average age of 21.92 years and a standard deviation of 1.77 years, with ages spanning from 18 to 25 years. Cam-CAN: This dataset includes individuals with a mean age of 54.33 years and a relatively high standard deviation of 18.30 years, reflecting a wide age range from 18 to 88 years. |
| Reporting on race, ethnicity, or other socially relevant groupings | There were no further socially relevant groupings utilised in this study. |
| Population characteristics | There were no further categorisations of population characteristics utilised in this study. |
| Recruitment | HCP-YA: The subject recruitment was organised and carried out by the WU-Minn HCP consortium. The project primarily recruited healthy individuals born in Missouri. Efforts were taken to make sure the ethnic and racial composition broadly reflected the 2000 decennial census. Individuals with severe neurodevelopmental disorders, documented neuropsychiatric disorders, or neurological disorders were excluded. The ID1000 dataset was collected between 2010 and 2012 after approval from the faculty's ethical committee (EC number: 2010-BC-1345). Data were recorded from 992 subjects, with 928 included after applying exclusion criteria such as MRI contraindications, aiming for a sample representative of the Dutch population in educational level (ages 19–26). The PIOP1 and PIOP2 datasets were collected between 2015–2016 and 2017 respectively, with ethical approval (PIOP1 EC: 2015-EXT-4366, PIOP2 EC: 2017-EXT-7568). Data were collected from 248 (PIOP1) and 242 (PIOP2) university students, with 216 and 226 included after exclusions. Subjects were recruited via university channels and social media. The CamCAN dataset included a three-stage protocol: Stage 1 collected home-based demographic, health, cognitive, and lifestyle data from 2681 adults; Stage 2 tested approximately 700 participants in a lab setting with MRI, MEG, cognitive tasks, and physiological measurements; Stage 3 followed up with around 280 participants for additional imaging, cognitive assessments, and health data within three years. |
| Ethics oversight | The data collection was approved by the respective local Institutional Review Board. Retrospective analysis of these datasets was further approved by the local Ethics Committee at the Faculty of Medicine at Heinrich-Heine-University in Düsseldorf. |

Note that full information on the approval of the study protocol must also be provided in the manuscript.

# Field-specific reporting

Please select the one below that is the best fit for your research. If you are not sure, read the appropriate sections before making your selection.

☒ Life sciences ☐ Behavioural & social sciences ☐ Ecological, evolutionary & environmental sciences

For a reference copy of the document with all sections, see nature.com/documents/nr-reporting-summary-flat.pdf

# Life sciences study design

All studies must disclose on these points even when the disclosure is negative.

| | |
|---|---|
| Sample size | HCP-YA (203 Male, 192 Female); PIOP1 (78 Male, 97 Female); PIOP2 (93 Male, 119 Female); Cam-CAN (312 Male, 315 Female) |
| Data exclusions | Participants were excluded if they lacked study critical imaging or behavioural measurements (e.g. fluid intelligence test score, rs-fMRI scans etc.) |
| Replication | We replicated findings across 4 distinct datasets (HCP-YA; AOMIC PIOP1 and PIOP2; Cam-CAN). |

| | |
|---|---|
| Randomization | Participants were not randomly assigned to experimental groups as we looked at individual differences and performed no group comparisons. Participants were randomly assigned to either a holdout or an analysis dataset using an 80:20 split. |
| Blinding | Not applicable. |

# Behavioural & social sciences study design

All studies must disclose on these points even when the disclosure is negative.

| | |
|---|---|
| Study description | *Briefly describe the study type including whether data are quantitative, qualitative, or mixed-methods (e.g. qualitative cross-sectional, quantitative experimental, mixed-methods case study).* |
| Research sample | *State the research sample (e.g. Harvard university undergraduates, villagers in rural India) and provide relevant demographic information (e.g. age, sex) and indicate whether the sample is representative. Provide a rationale for the study sample chosen. For studies involving existing datasets, please describe the dataset and source.* |
| Sampling strategy | *Describe the sampling procedure (e.g. random, snowball, stratified, convenience). Describe the statistical methods that were used to predetermine sample size OR if no sample-size calculation was performed, describe how sample sizes were chosen and provide a rationale for why these sample sizes are sufficient. For qualitative data, please indicate whether data saturation was considered, and what criteria were used to decide that no further sampling was needed.* |
| Data collection | *Provide details about the data collection procedure, including the instruments or devices used to record the data (e.g. pen and paper, computer, eye tracker, video or audio equipment) whether anyone was present besides the participant(s) and the researcher, and whether the researcher was blind to experimental condition and/or the study hypothesis during data collection.* |
| Timing | *Indicate the start and stop dates of data collection. If there is a gap between collection periods, state the dates for each sample cohort.* |
| Data exclusions | *If no data were excluded from the analyses, state so OR if data were excluded, provide the exact number of exclusions and the rationale behind them, indicating whether exclusion criteria were pre-established.* |
| Non-participation | *State how many participants dropped out/declined participation and the reason(s) given OR provide response rate OR state that no participants dropped out/declined participation.* |
| Randomization | *If participants were not allocated into experimental groups, state so OR describe how participants were allocated to groups, and if allocation was not random, describe how covariates were controlled.* |

# Ecological, evolutionary & environmental sciences study design

All studies must disclose on these points even when the disclosure is negative.

| | |
|---|---|
| Study description | *Briefly describe the study. For quantitative data include treatment factors and interactions, design structure (e.g. factorial, nested, hierarchical), nature and number of experimental units and replicates.* |
| Research sample | *Describe the research sample (e.g. a group of tagged Passer domesticus, all Stenocereus thurberi within Organ Pipe Cactus National Monument), and provide a rationale for the sample choice. When relevant, describe the organism taxa, source, sex, age range and any manipulations. State what population the sample is meant to represent when applicable. For studies involving existing datasets, describe the data and its source.* |
| Sampling strategy | *Note the sampling procedure. Describe the statistical methods that were used to predetermine sample size OR if no sample-size calculation was performed, describe how sample sizes were chosen and provide a rationale for why these sample sizes are sufficient.* |
| Data collection | *Describe the data collection procedure, including who recorded the data and how.* |
| Timing and spatial scale | *Indicate the start and stop dates of data collection, noting the frequency and periodicity of sampling and providing a rationale for these choices. If there is a gap between collection periods, state the dates for each sample cohort. Specify the spatial scale from which the data are taken* |
| Data exclusions | *If no data were excluded from the analyses, state so OR if data were excluded, describe the exclusions and the rationale behind them, indicating whether exclusion criteria were pre-established.* |
| Reproducibility | *Describe the measures taken to verify the reproducibility of experimental findings. For each experiment, note whether any attempts to repeat the experiment failed OR state that all attempts to repeat the experiment were successful.* |
| Randomization | *Describe how samples/organisms/participants were allocated into groups. If allocation was not random, describe how covariates were controlled. If this is not relevant to your study, explain why.* |
| Blinding | *Describe the extent of blinding used during data acquisition and analysis. If blinding was not possible, describe why OR explain why blinding was not relevant to your study.* |

Did the study involve field work? ☐ Yes ☒ No

## Field work, collection and transport

| | |
|---|---|
| Field conditions | *Describe the study conditions for field work, providing relevant parameters (e.g. temperature, rainfall).* |
| Location | *State the location of the sampling or experiment, providing relevant parameters (e.g. latitude and longitude, elevation, water depth).* |
| Access & import/export | *Describe the efforts you have made to access habitats and to collect and import/export your samples in a responsible manner and in compliance with local, national and international laws, noting any permits that were obtained (give the name of the issuing authority, the date of issue, and any identifying information).* |
| Disturbance | *Describe any disturbance caused by the study and how it was minimized.* |

# Reporting for specific materials, systems and methods

We require information from authors about some types of materials, experimental systems and methods used in many studies. Here, indicate whether each material, system or method listed is relevant to your study. If you are not sure if a list item applies to your research, read the appropriate section before selecting a response.

### Materials & experimental systems

| n/a | Involved in the study |
|---|---|
| ☒ | ☐ Antibodies |
| ☒ | ☐ Eukaryotic cell lines |
| ☒ | ☐ Palaeontology and archaeology |
| ☒ | ☐ Animals and other organisms |
| ☒ | ☐ Clinical data |
| ☒ | ☐ Dual use research of concern |
| ☒ | ☐ Plants |

### Methods

| n/a | Involved in the study |
|---|---|
| ☒ | ☐ ChIP-seq |
| ☒ | ☐ Flow cytometry |
| ☐ | ☒ MRI-based neuroimaging |

## Antibodies

| | |
|---|---|
| Antibodies used | *Describe all antibodies used in the study; as applicable, provide supplier name, catalog number, clone name, and lot number.* |
| Validation | *Describe the validation of each primary antibody for the species and application, noting any validation statements on the manufacturer's website, relevant citations, antibody profiles in online databases, or data provided in the manuscript.* |

## Eukaryotic cell lines

Policy information about cell lines and Sex and Gender in Research

| | |
|---|---|
| Cell line source(s) | *State the source of each cell line used and the sex of all primary cell lines and cells derived from human participants or vertebrate models.* |
| Authentication | *Describe the authentication procedures for each cell line used OR declare that none of the cell lines used were authenticated.* |
| Mycoplasma contamination | *Confirm that all cell lines tested negative for mycoplasma contamination OR describe the results of the testing for mycoplasma contamination OR declare that the cell lines were not tested for mycoplasma contamination.* |
| Commonly misidentified lines (See ICLAC register) | *Name any commonly misidentified cell lines used in the study and provide a rationale for their use.* |

## Palaeontology and Archaeology

| | |
|---|---|
| Specimen provenance | *Provide provenance information for specimens and describe permits that were obtained for the work (including the name of the issuing authority, the date of issue, and any identifying information). Permits should encompass collection and, where applicable, export.* |
| Specimen deposition | *Indicate where the specimens have been deposited to permit free access by other researchers.* |
| Dating methods | *If new dates are provided, describe how they were obtained (e.g. collection, storage, sample pretreatment and measurement), where* |

| | |
|---|---|
| Dating methods | *they were obtained (i.e. lab name), the calibration program and the protocol for quality assurance OR state that no new dates are provided.* |

☐ Tick this box to confirm that the raw and calibrated dates are available in the paper or in Supplementary Information.

| | |
|---|---|
| Ethics oversight | *Identify the organization(s) that approved or provided guidance on the study protocol, OR state that no ethical approval or guidance was required and explain why not.* |

Note that full information on the approval of the study protocol must also be provided in the manuscript.

# Animals and other research organisms

Policy information about studies involving animals; ARRIVE guidelines recommended for reporting animal research, and Sex and Gender in Research

| | |
|---|---|
| Laboratory animals | *For laboratory animals, report species, strain and age OR state that the study did not involve laboratory animals.* |
| Wild animals | *Provide details on animals observed in or captured in the field; report species and age where possible. Describe how animals were caught and transported and what happened to captive animals after the study (if killed, explain why and describe method; if released, say where and when) OR state that the study did not involve wild animals.* |
| Reporting on sex | *Indicate if findings apply to only one sex; describe whether sex was considered in study design, methods used for assigning sex. Provide data disaggregated for sex where this information has been collected in the source data as appropriate; provide overall numbers in this Reporting Summary. Please state if this information has not been collected. Report sex-based analyses where performed, justify reasons for lack of sex-based analysis.* |
| Field-collected samples | *For laboratory work with field-collected samples, describe all relevant parameters such as housing, maintenance, temperature, photoperiod and end-of-experiment protocol OR state that the study did not involve samples collected from the field.* |
| Ethics oversight | *Identify the organization(s) that approved or provided guidance on the study protocol, OR state that no ethical approval or guidance was required and explain why not.* |

Note that full information on the approval of the study protocol must also be provided in the manuscript.

# Clinical data

Policy information about clinical studies

All manuscripts should comply with the ICMJE guidelines for publication of clinical research and a completed CONSORT checklist must be included with all submissions.

| | |
|---|---|
| Clinical trial registration | *Provide the trial registration number from ClinicalTrials.gov or an equivalent agency.* |
| Study protocol | *Note where the full trial protocol can be accessed OR if not available, explain why.* |
| Data collection | *Describe the settings and locales of data collection, noting the time periods of recruitment and data collection.* |
| Outcomes | *Describe how you pre-defined primary and secondary outcome measures and how you assessed these measures.* |

# Dual use research of concern

Policy information about dual use research of concern

## Hazards

Could the accidental, deliberate or reckless misuse of agents or technologies generated in the work, or the application of information presented in the manuscript, pose a threat to:

| No | Yes | |
|---|---|---|
| ☐ | ☐ | Public health |
| ☐ | ☐ | National security |
| ☐ | ☐ | Crops and/or livestock |
| ☐ | ☐ | Ecosystems |
| ☐ | ☐ | Any other significant area |

## Experiments of concern

Does the work involve any of these experiments of concern:

| No | Yes | |
|---|---|---|
| ☐ | ☐ | Demonstrate how to render a vaccine ineffective |
| ☐ | ☐ | Confer resistance to therapeutically useful antibiotics or antiviral agents |
| ☐ | ☐ | Enhance the virulence of a pathogen or render a nonpathogen virulent |
| ☐ | ☐ | Increase transmissibility of a pathogen |
| ☐ | ☐ | Alter the host range of a pathogen |
| ☐ | ☐ | Enable evasion of diagnostic/detection modalities |
| ☐ | ☐ | Enable the weaponization of a biological agent or toxin |
| ☐ | ☐ | Any other potentially harmful combination of experiments and agents |

# Plants

| | |
|---|---|
| Seed stocks | *Report on the source of all seed stocks or other plant material used. If applicable, state the seed stock centre and catalogue number. If plant specimens were collected from the field, describe the collection location, date and sampling procedures.* |
| Novel plant genotypes | *Describe the methods by which all novel plant genotypes were produced. This includes those generated by transgenic approaches, gene editing, chemical/radiation-based mutagenesis and hybridization. For transgenic lines, describe the transformation method, the number of independent lines analyzed and the generation upon which experiments were performed. For gene-edited lines, describe the editor used, the endogenous sequence targeted for editing, the targeting guide RNA sequence (if applicable) and how the editor was applied.* |
| Authentication | *Describe any authentication procedures for each seed stock used or novel genotype generated. Describe any experiments used to assess the effect of a mutation and, where applicable, how potential secondary effects (e.g. second site T-DNA insertions, mosiacism, off-target gene editing) were examined.* |

# ChIP-seq

## Data deposition

☐ Confirm that both raw and final processed data have been deposited in a public database such as GEO.

☐ Confirm that you have deposited or provided access to graph files (e.g. BED files) for the called peaks.

| | |
|---|---|
| Data access links<br>*May remain private before publication.* | *For "Initial submission" or "Revised version" documents, provide reviewer access links. For your "Final submission" document, provide a link to the deposited data.* |
| Files in database submission | *Provide a list of all files available in the database submission.* |
| Genome browser session<br>(e.g. UCSC) | *Provide a link to an anonymized genome browser session for "Initial submission" and "Revised version" documents only, to enable peer review. Write "no longer applicable" for "Final submission" documents.* |

## Methodology

| | |
|---|---|
| Replicates | *Describe the experimental replicates, specifying number, type and replicate agreement.* |
| Sequencing depth | *Describe the sequencing depth for each experiment, providing the total number of reads, uniquely mapped reads, length of reads and whether they were paired- or single-end.* |
| Antibodies | *Describe the antibodies used for the ChIP-seq experiments; as applicable, provide supplier name, catalog number, clone name, and lot number.* |
| Peak calling parameters | *Specify the command line program and parameters used for read mapping and peak calling, including the ChIP, control and index files used.* |
| Data quality | *Describe the methods used to ensure data quality in full detail, including how many peaks are at FDR 5% and above 5-fold enrichment.* |
| Software | *Describe the software used to collect and analyze the ChIP-seq data. For custom code that has been deposited into a community repository, provide accession details.* |

# Flow Cytometry

## Plots

Confirm that:

- [ ] The axis labels state the marker and fluorochrome used (e.g. CD4-FITC).
- [ ] The axis scales are clearly visible. Include numbers along axes only for bottom left plot of group (a 'group' is an analysis of identical markers).
- [ ] All plots are contour plots with outliers or pseudocolor plots.
- [ ] A numerical value for number of cells or percentage (with statistics) is provided.

## Methodology

| | |
|---|---|
| Sample preparation | *Describe the sample preparation, detailing the biological source of the cells and any tissue processing steps used.* |
| Instrument | *Identify the instrument used for data collection, specifying make and model number.* |
| Software | *Describe the software used to collect and analyze the flow cytometry data. For custom code that has been deposited into a community repository, provide accession details.* |
| Cell population abundance | *Describe the abundance of the relevant cell populations within post-sort fractions, providing details on the purity of the samples and how it was determined.* |
| Gating strategy | *Describe the gating strategy used for all relevant experiments, specifying the preliminary FSC/SSC gates of the starting cell population, indicating where boundaries between "positive" and "negative" staining cell populations are defined.* |

- [ ] Tick this box to confirm that a figure exemplifying the gating strategy is provided in the Supplementary Information.

# Magnetic resonance imaging

## Experimental design

| | |
|---|---|
| Design type | Analysis of individual differences using identification paradigm and machine learning workflow. |
| Design specifications | *Specify the number of blocks, trials or experimental units per session and/or subject, and specify the length of each trial or block (if trials are blocked) and interval between trials.* |
| Behavioral performance measures | fluid intelligence (Raven's Matrices and Cattell Culture Fair Intelligence Test) |

## Acquisition

| | |
|---|---|
| Imaging type(s) | fMRI |
| Field strength | HCP-YA (3T); AOMIC PIOP1 and PIOP2 (3T); Cam-CAN (3T) |
| Sequence & imaging parameters | HCP.YA (Scans were acquired using a 3T Siemens connectome-Skyra scanner with a gradient-echo EPI sequence (TE=33.1ms, TR=720ms, flip angle = 52°, 2.0mm isotropic voxels, 72 slices, multiband factor of 8). ); AOMIC PIOP1 and PIOP2 (The functional MRI scanning protocol for the PIOP1 and PIOP2 data sets utilised a Philips 3T scanner, with PIOP1 scanned on the "Achieva'' version and PIOP2 on the "Achieva dStream" version, both equipped with a 32-channel head coil. Functional MRI data in PIOP1 for resting-state fMRI were acquired with multiband acceleration, whereas functional MRI scans in the PIOP2 were acquired sequentially. For both data sets, resting state scans lasted ~6 minutes (PIOP1; i.e., 480 volumes with a 0.75 second TR) and ~8 minutes (PIOP2; i.e., 240 volumes with a 2 second TR).); Cam-CAN (Resting state fMRI data in the Cam-CAN dataset were acquired using a T2*-weighted Gradient-Echo Echo-Planar Imaging (EPI) sequence while participants rested with their eyes closed. A total of 261 volumes were collected, each comprising 32 axial slices acquired in descending order. The slices had a thickness of 3.7 mm with an interslice gap of 20%, ensuring whole-brain coverage including the cerebellum. The acquisition parameters included a repetition time (TR) of 1970 milliseconds, an echo time (TE) of 30 milliseconds, a flip angle of 78 degrees, and a field of view (FOV) of 192 mm × 192 mm. The voxel size was 3 mm × 3 mm × 4.44 mm, and the total acquisition time for each scan was 8 minutes and 40 seconds.<br>) |
| Area of acquisition | Whole Brain |

Diffusion MRI ☐ Used ☒ Not used

## Preprocessing

| | |
|---|---|
| Preprocessing software | fMRIPrep, HCP Minimal Pipeline; FSL ICA-FIX; Junifer |

| | |
|---|---|
| Normalization | Spatial normalisation using non-linear registration to FSL's MNI template in the HCP-YA dataset. Spatial normalisation to the ICBM 152 Nonlinear Asymmetrical template was performed using nonlinear registration wialisation Spatial normalisation to the ICBM 152 Nonlinear Asymmetrical template was performed using nonlinear th ANTs in other datasets. |
| Normalization template | MNI152 |
| Noise and artifact removal | ICA FIX + nuisance regression in HCP-YA; fMRIPRep and nuisance regression in other datasets |
| Volume censoring | No censoring performed. |

## Statistical modeling & inference

| | |
|---|---|
| Model type and settings | Ridge regression; For the ridge classifier we tuned the “alpha” parameter using a grid generated by numpy (Harris et al., 2020) as “np.geomspace(0.001, 10000, 50)”. |
| Effect(s) tested | Prediction of fluid intelligence, age, head motion in scanner |

Specify type of analysis: ☒ Whole brain ☐ ROI-based ☐ Both

| | |
|---|---|
| Statistic type for inference (See Eklund et al. 2016) | No inferential analysis performed. Predictive analysis. |
| Correction | *Describe the type of correction and how it is obtained for multiple comparisons (e.g. FWE, FDR, permutation or Monte Carlo).* |

## Models & analysis

| n/a | Involved in the study |
|---|---|
| ☐ | ☒ Functional and/or effective connectivity |
| ☒ | ☐ Graph analysis |
| ☐ | ☒ Multivariate modeling or predictive analysis |

| | |
|---|---|
| Functional and/or effective connectivity | Functional Connectivity derived features were used in the prediction workflows. |
| Graph analysis | None performed. |
| Multivariate modeling and predictive analysis | Prediction of fluid intelligence, age, head motion in scanner |
